# Ferroptosis in pulmonary fibrosis: pathogenesis and traditional Chinese medicine-driven therapeutic approaches

**DOI:** 10.3389/fcell.2025.1598924

**Published:** 2025-07-18

**Authors:** Xudong Fan, Jiangxin Xu, Jinlong Gao, Jie Zhang, Yu Wang, Yanni Shan, Jingming Luo, Weidong Fei, Xinjun Cai

**Affiliations:** ^1^Department of Pharmacy, Zhejiang Hospital of Integrated Traditional Chinese and Western Medicine, Hangzhou, China; ^2^School of Pharmacy, Hangzhou Normal University, Hangzhou, China; ^3^School of Pharmaceutical Sciences, Zhejiang Chinese Medical University, Hangzhou, China; ^4^Women’s Hospital, Zhejiang University School of Medicine, Hangzhou, China

**Keywords:** pulmonary fibrosis, ferroptosis, traditional Chinese medicine, nanotechnology, therapy

## Abstract

Pulmonary fibrosis (PF) is a progressive interstitial lung disease marked by the excessive buildup of fibrous connective tissue, leading to permanent damage to respiratory function due to irreversible changes in lung structure. Despite significant progress in understanding its underlying mechanisms, translating this knowledge into effective prevention or treatment remains a major clinical challenge. Ferroptosis, a form of controlled cellular demise triggered by iron, involves the accumulation of lipid peroxides, resulting in irreversible membrane disintegration and oxidative metabolic failure. Emerging studies suggest that ferroptosis exacerbates PF progression by promoting macrophage polarization, fibroblast proliferation, and extracellular matrix deposition, ultimately leading to alveolar epithelial cell death and fibrotic tissue remodeling. Consequently, targeting ferroptosis presents a promising therapeutic approach, with traditional Chinese medicine (TCM) showing particular potential through its multi-dimensional and holistic mechanisms. TCM compounds, extracts, and bioactive monomers exhibit anti-inflammatory, antioxidant, and multi-target properties that demonstrate significant value in managing PF. To develop innovative therapeutic strategies for PF, this review synthesizes recent progress in elucidating ferroptosis pathways implicated in the pathogenesis of PF and underscores the therapeutic potential of TCM in PF management via ferroptosis inhibition. Moreover, this paper highlights the advantages of integrating nanotechnology with TCM for regulating ferroptosis in PF treatment. In general, this paper will provide new perspectives for advancing research and clinical applications of TCM in the treatment of PF.

## 1 Introduction

Pulmonary fibrosis (PF) is a chronic and progressive pulmonary disorder marked by the proliferation of fibrous tissue and the formation of scar tissue within the lung parenchyma. This condition impairs lung function and reduces the oxygen supply to the body, leading to severe respiratory issues and potentially fatal complications (Ma et al., 2024). Idiopathic pulmonary fibrosis is the most common form of PF. Epidemiological studies indicate that the global incidence of idiopathic pulmonary fibrosis ranges from 0.09 to 1.30 cases per 10,000 annually and is on the rise (Maher et al., 2021; Hu et al., 2024). The primary clinical treatments for PF include pharmacotherapy (pirfenidone and nindazanib), oxygen therapy, and lung transplantation (Ma et al., 2024). Despite ongoing research into its causes and mechanisms, PF remains a significant clinical challenge, as there are no therapies available that can reverse the disease. Prognosis remains poor, with a median survival time of less than 5 years for most patients (Meyer, 2017; Savin et al., 2022). Therefore, the vigorous development of anti-fibrotic treatments is of great clinical importance.

Ferroptosis, an iron-dependent form of regulated cell death, is characterized by an excessive buildup of lipid peroxides and disrupted redox homeostasis (Jiang et al., 2021; Xu et al., 2021). The principal mechanisms involve dysregulated iron metabolism, generation of reactive oxygen species (ROS), peroxidation of polyunsaturated fatty acids (PUFAs), glutathione (GSH) depletion, and inhibition of glutathione peroxidase 4 (GPX4) (Dixon et al., 2012; Hu et al., 2024). Morphologically, ferroptotic cells display a distinct ballooning phenotype, characterized by clear, rounded contours and a translucent, vacuolized cytosol. Additionally, their mitochondria are shrunken with reduced or absent cristae, features that distinguish this process from apoptosis, necrosis, and autophagy (Jiang et al., 2021; Anna Martina et al., 2023; Alessandro et al., 2024; Zhang F. et al., 2024). Ferroptosis is closely associated with the pathology of various diseases, acting as a “double-edged sword” by either promoting disease progression or serving as a therapeutic target. A large number of recent studies have found a high correlation between ferroptosis and PF. Through mechanisms such as lipid peroxidation and oxidative stress, ferroptosis plays a crucial role in the occurrence and development of PF (Pei et al., 2022; Yang H. H. et al., 2022; Sun L. F. et al., 2024). Recent studies have increasingly explored the potential of traditional Chinese medicine (TCM) in mitigating PF through the regulation of ferroptosis (Chen T. et al., 2024).

**FIGURE 1:**
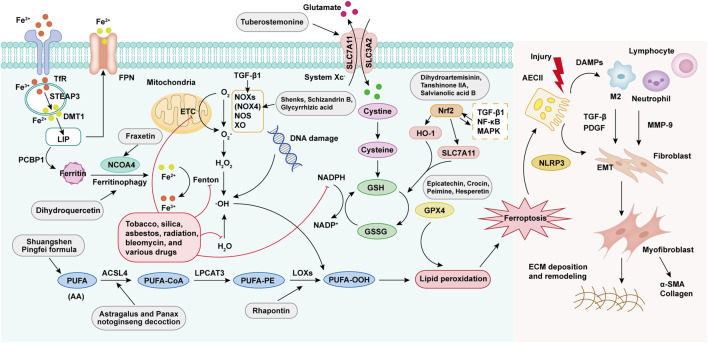
Molecular mechanisms of ferroptosis in PF progression. Ferroptosis is activated through four core pathways: (1) Iron metabolism dysregulation causes intracellular free iron accumulation, facilitating Fenton reactions; (2) Overproduction of ROS mediated by mitochondria and NADPH oxidase; (3) Peroxidation of PUFAs via LOX activity; (4) Dysfunction of the GPX4-dependent antioxidant system. These mechanisms collectively result in lipid peroxidation, leading to ferroptosis. Damaged AECIIs release damage-associated molecular patterns (DAMPs), attracting and activating immune cells (e.g., macrophages, neutrophils, lymphocytes), which secrete pro-fibrotic factors including TGF-β, platelet-derived growth factor (PDGF), and matrix metalloproteinase-9 (MMP-9). These signals activate the epithelial-mesenchymal transformation (EMT) program, which promotes fibroblast proliferation/differentiation, leading to abnormal collagen deposition and tissue remodeling in the extracellular matrix (ECM), ultimately contributing to PF.

To explore new therapeutic strategies, the first part of this review elaborates on the relationship between ferroptosis and the development of PF (Figure 1). TCM, with its multi-component, multi-target, and multi-pathway advantages, presents a promising approach. Herbal medicines and other naturally derived active compounds possess anti-inflammatory, antioxidant, anti-tumor, and immunomodulatory effects, holding significant value in the prevention and treatment of PF (Chen Y. Q. et al., 2024; Xu et al., 2024). Our reviewed literature indicates that targeting ferroptosis is a crucial mechanism for treating PF with TCM. The second part of this review summarizes the existing evidence supporting the use of TCM to modulate ferroptosis in managing PF, highlighting specific TCMs and their active compounds that demonstrate anti-fibrotic potential through ferroptosis modulation (Figure 1). Furthermore, nanotechnology-enabled delivery systems (e.g., liposomes, polymeric nanoparticles) enhance TCM bioavailability, enable targeted accumulation in fibrotic lesions, and reduce off-target toxicity of TCM. This paper also analyzes the application of nanotechnology-assisted TCM delivery in reversing ferroptosis-mediated PF, as well as biomedical engineering technology. Finally, the challenges and future prospects of TCM-based therapeutic strategies targeting ferroptosis inhibition are discussed. In summary, this review synthesizes recent research on the role of TCM in PF treatment, focusing on its effects on ferroptosis and related signaling pathways, to guide TCM-based strategies for fibrotic diseases.

## 2 Role of ferroptosis in PF development

### 2.1 Abnormal iron metabolism

Iron is an essential trace element in human physiology, playing a vital role in the regulation of systemic biological processes (Pei et al., 2022). Under normal conditions, pulmonary iron homeostasis is maintained by macrophage phagocytosis, epithelial antioxidant defenses, and the mucociliary clearance system. Additionally, the lung employs a specific detoxification process by releasing transferrin and ferritin into the epithelial lining fluid. These iron-binding proteins are either cleared by the mucociliary escalator or sequestered long-term in the reticuloendothelial system, thus preventing iron-induced oxidative stress (Tomas and Elizabeta, 2015; Bruno et al., 2023). Recent studies indicate that disruptions in iron metabolism in the lungs are closely linked to the onset and progression of PF (Yuan et al., 2022; Zhai et al., 2023). These metabolic disturbances arise from disorders in iron absorption, transport, storage, and utilization, influenced by various external factors, diet, intestinal function, genetic predispositions, abnormalities in key proteins, changes in iron storage forms, and iron utilization disorders, all of which interact to contribute to PF (Li et al., 2021b). Redox-active iron, particularly Fe^2+^, facilitates hydroxyl radical production via the Fenton reaction, exacerbating ROS-induced tissue damage, inflammation, and lipid peroxidation, thereby promoting fibrosis and lung function decline (Dixon and Stockwell, 2014). In animals with PF, lung iron metabolism abnormalities, characterized by increased iron levels, iron-laden macrophages, and oxidative stress induced by iron, may contribute to the development and progression of PF (Sun Y. et al., 2024). In a study, Shao et al. found that in a bleomycin (BLM)-induced PF model, mitochondrial iron deposition in alveolar epithelial type II cells (AECIIs) increase significantly, leading to mitochondrial dysfunction and cellular damage, with the upregulation of the mitochondrial iron transporter Mitoferrin-2 being a key factor (Shao et al., 2024). In PF, abnormal iron metabolism is closely related to macrophage irregularities, jointly promoting disease progression. Studies have found that in the BLM-induced PF mouse model, Tfr1^+^ macrophages increase and display an M2 phenotype, but decrease following treatment with the iron chelator deferoxamine (Ali et al., 2020; Ogger and Byrne, 2020). Importantly, systemic iron overload leads to excessive iron deposition in lung cells, particularly in AECIIs, alveolar macrophages, vascular smooth muscle cells, and ciliated airway epithelial cells (Figure 1) (Neves et al., 2017). Overall, maintaining iron homeostasis is critical for lung health, as its disruption can initiate fibrotic processes. Targeting iron metabolism pathways offers a promising therapeutic approach for PF intervention.

### 2.2 ROS generation

The lungs are especially vulnerable to oxidative stress compared to other organs due to their direct exposure to high oxygen levels (Cheresh et al., 2013). They are regularly exposed to reactive oxidants from external sources such as tobacco, asbestos/silica, radiation, bleomycin, and various drugs, as well as from internal sources produced by inflammatory cells, and epithelial, mesenchymal, and endothelial cells within tissues. Several enzymatic systems, including nicotinamide adenine dinucleotide phosphate (NADPH) oxidases (NOXs), xanthine oxidase (XO), nitric oxide synthase (NOS), and the mitochondrial electron transport chain, contribute to ROS production (Winterbourn, 2008). Most ferroptosis-associated ROS stem from the Fenton and Haber-Weiss reactions (Jiang et al., 2024). Excessive ROS in the lung can trigger lipid peroxidation. ROS interact with PUFAs in lipid membranes, forming lipid peroxides that, when present in large amounts, can cause ferroptosis (Endale et al., 2023). Increased ROS production is a key factor in PF, contributing to epithelial cell death and fibroblast differentiation, leading to DNA damage and telomere shortening, which are indicative of the disease (Kliment and Oury, 2010; McDonough et al., 2018). Transforming growth factor-β1 (TGF-β1) stimulates fibrotic responses in lung epithelial cells through NADPH oxidase 4 (NOX4)-mediated activation of the SRC kinase FYN, which then induces mitochondrial ROS generation, DNA damage responses, and the expression of profibrotic genes (Veith et al., 2021). NOX4 plays a crucial role in regulating the pulmonary myofibroblast phenotype in PF, acting as an important regulator of Smad2/3 transcriptional activation downstream of TGF-β1 signaling in pulmonary fibroblasts (Zhang et al., 2017). In addition, ROS-driven PF is positively correlated with cellular senescence, a process that may be exacerbated by the activation of the NOD-like receptor thermal protein domain-associated protein 3 (NLRP3) inflammasome (Feng et al., 2024). Excessive ROS production exacerbates PF by inducing oxidative stress, alveolar epithelial cell injury, and pro-inflammatory signaling (Figure 1). Therapeutic approaches, such as antioxidant agents, ROS-scavenging enzymes, or targeted inhibition of NOX4-mediated ROS generation, may mitigate pathological remodeling and restore redox homeostasis, offering new methods to halt PF progression.

### 2.3 PUFAs peroxidation

The excessive oxidation of phospholipids containing PUFAs is considered a key feature of ferroptosis (Wiernicki et al., 2020). PUFAs, serving as the primary substrates for lipid metabolism in ferroptosis, especially arachidonic acid (AA) and adrenaline, are converted into PUFA-PE through the action of acyl-CoA synthetase long chain member 4 (ACSL4) and lysophosphatidylcholine acyltransferase 3 (LPCAT3). Once oxidized by lipoxygenases (LOXs), these products can generate lipid peroxides like polyunsaturated fatty acid hydroperoxide (PUFA-OOH) (Liang et al., 2022). During AA metabolism, inflammatory mediators such as leukotrienes are produced, which have strong chemotactic properties that attract inflammatory cells such as macrophages and neutrophils to the lung tissue, triggering or worsening the inflammatory response. This persistent inflammation forms a crucial pathological basis for the occurrence and progression of PF (Figure 1) (Charbeneau and Peters-Golden, 2005; Chen and Dai, 2023). Chung et al. found an increase in the expression of 12-LOX within a radiation-induced mouse model of PF. This change promotes the metabolism of more PUFAs and simultaneously stimulates type II pneumocytes to secrete interleukin-4 (IL-4) and interleukin-13 (IL-13). In the inflammatory microenvironment of the lung tissue, various cytokines and ROS produced can, in turn, influence the activity and expression of enzymes related to PUFAs metabolism. For example, in a paraquat-induced mouse model of PF, Tomitsuka et al. identified not only the upregulation of inflammation-related genes but also an increase in ACSL4 expression in alveolar epithelial cells. This upregulation further exacerbates ferroptosis and the inflammatory response, establishing a vicious cycle that accelerates the progression of PF (Chung et al., 2019). Therefore, targeting PUFA peroxidation may present a promising new approach for treating PF.

### 2.4 Imbalance of antioxidant system

Excessive ROS production and weakened antioxidant defenses together lead to increased oxidative stress, which is mechanistically associated with the progression of PF (Hu et al., 2024). Antioxidants play a vital role in mitigating oxidative stress by donating hydrogen atoms, thereby interrupting the peroxidation chain reaction. Multiple pathways, such as the actions of catalase (CAT), superoxide dismutase (SOD), GSH, and GPX4, contribute to the inhibition of lipid peroxidation (Liang et al., 2023; von Krusenstiern et al., 2023). In one study, Shariati et al. reported a reduction in the activities of SOD, CAT, and GPX enzymes, along with decreased GSH levels and increased malondialdehyde (MDA) levels in BLM-induced pulmonary fibrotic tissues (Shariati et al., 2019). Furthermore, another study suggested that downregulation of solute carrier family 7 member 11 (SLC7A11, a component of the cystine/glutamate antiporter) can suppress GPX4 activity by disrupting cystine metabolism. This leads to the accumulation of lipid peroxides and PF development (Liu et al., 2024). Mitogen-activated protein kinases (MAPKs), including extracellular signal-regulated kinase (ERK), c-Jun N-terminal kinase (JNK), and p38, are activated by oxidative stress through phosphorylation. Additionally, MAPKs can activate nuclear factor kappa-B (NF-κB), with the MAPK/NF-κB pathway playing a crucial role in PF pathogenesis (Zhu et al., 2024).

The nuclear factor erythroid 2-related factor 2 (Nrf2) is an essential transcription factor integral to the cellular response to oxidative stress (Dodson et al., 2019). Under normal conditions, Nrf2 and its target genes are expressed at basal levels (Zaghloul et al., 2019; Li et al., 2025). However, during the development of PF, reduced Nrf2 expression is strongly linked to oxidative stress imbalance. This imbalance may lead to continuous damage to AECIIs, subsequently triggering excessive activation and repair dysregulation of myofibroblasts in lung tissue, which ultimately results in the onset of PF (Figure 1) (Yang et al., 2018). Therefore, dysregulation of Nrf2 activity has been proposed as a contributing factor in the pathogenesis of PF.

## 3 TCM for treating PF through ferroptosis regulatory pathway

### 3.1 Regulation of iron metabolism

Ferritinophagy is a process in which nuclear receptor coactivator 4 (NCOA4) selectively binds to ferritin and facilitates its degradation in lysosomes. This action increases intracellular iron content and the labile iron pool (LIP), triggering ferroptosis (Gao et al., 2016). Inhibitors of ferritinophagy and NCOA4 can block ferritin degradation, prevent erastin-mediated ferroptosis, and mitigate PF. Yuan et al. investigated how dihydroquercetin (DHQ) alleviates silica-induced PF by inhibiting the ferroptosis signaling pathway. Their research demonstrated that DHQ significantly reduces inflammation and fibrosis in lung tissues in both *in vivo* and *in vitro* experiments. DHQ hinders the onset of ferroptosis by lowering iron accumulation and lipid peroxidation products, while boosting GPX4 and GSH levels. In addition, DHQ inhibits ferritinophagy by downregulating the expression of microtubule-associated protein 1A/1B-light chain 3 and upregulating the expression of ferritin heavy chain 1 and NCOA4, thereby further suppressing ferroptosis. Animal experiments revealed that DHQ treatment notably alleviates silica-induced PF in mice, reduces collagen deposition and inflammation, and lowers pro-inflammatory cytokine levels (Yuan et al., 2022). Hence, DHQ exhibits notable advantages in mitigating PF through multiple mechanisms. Its efficacy in both cellular and animal models underscores its potential as a natural therapeutic agent for silica-induced PF, offering a multi-target approach with reduced toxicity risks compared to synthetic drugs.

Building on this, recent research highlights additional natural compounds and formulations that similarly regulate iron metabolism to counteract PF. The natural product fraxetin effectively suppresses ferroptosis by decreasing NCOA4 expression, thereby providing protection against pulmonary inflammation and fibrosis in BLM-induced PF mouse models (Zhai et al., 2023). As a phytochemical, fraxetin boasts a favorable safety profile with potentially lower toxicity compared to synthetic drugs. Its multi-target mechanism includes suppressing iron overload and ferroptosis by downregulating NCOA4, thus maintaining alveolar epithelial cell integrity. Additionally, fraxetin mitigates inflammation by reducing pro-inflammatory cytokine release and enhances mitochondrial function, addressing key pathological factors of fibrosis. Unlike current therapies like nintedanib and pirfenidone, which primarily slow disease progression, fraxetin’s dual action on ferroptosis and inflammation highlights its promise as a novel, naturally derived therapeutic agent for PF. Another study revealed that the Qingfei Xieding prescription (QF) lowered Fe^2+^ levels, decreased the mortality rate of mice, alleviated the inflammation and fibrosis of lung tissues, and improved lung function (Sun Y. et al., 2024). QF’s synergistic composition targets multiple pathways, including inhibition of ferroptosis and modulation of the ACE2-ERK signaling axis, resulting in reduced lipid peroxidation, iron overload, and mitochondrial damage. Unlike single-target therapies, QF’s holistic action combines anti-fibrotic, anti-inflammatory, and antioxidant effects, underscoring its potential as a safer, naturally derived alternative with broader therapeutic efficacy for PF.

### 3.2 Inhibition of ROS generation

Oxidation plays a significant role in fibrogenesis by causing oxidative damage to critical biomolecules such as DNA, lipids, and proteins, primarily due to ROS overproduction (Cheresh et al., 2013). Recent studies emphasize the crucial role of NOX family oxidoreductases in maintaining redox homeostasis through enzymatic ROS generation during fibrogenesis (Hecker et al., 2012). Specifically, NOX4 is identified as a key regulator of myofibroblast activation within PF microenvironments (Amara et al., 2010). These mechanistic insights have led to the exploration of NOX isoform-specific inhibition as a promising therapeutic approach to mitigate pathological ECM remodeling in fibrotic diseases. Chu et al. investigated the anti-fibrotic mechanisms of Shen-mai-kai-fei-san (Shenks) on PF. Both prophylactic and therapeutic administration of Shenks significantly reduces BLM-induced PF in C57BL/6 female mice, decreasing lung collagen content and mRNA levels of Col1a1, Col1a2, Col3a1, connective tissue growth factor (CTGF), and TGF-β. Furthermore, Shenks lowers the number of inflammatory cells in bronchoalveolar lavage fluid. Mechanistically, it blocks the TGF-β pathway by reducing Smad3 phosphorylation and Smad-binding element activity. Notably, Shenks inhibits NOX4 expression and ROS production while upregulating antioxidant genes. These findings indicate that Shenks can inhibit PF, highlighting its potential as a treatment for this disease (Chu et al., 2017). As a TCM formula, Shenks offers unique benefits in combating PF through a holistic and synergistic approach based on TCM principles. It comprises multiple herbs classified under the “Jun-Chen-Zuo-Shi” framework, balancing the body’s Qi and Yin-Yang while addressing both root causes and symptoms. Yang et al. reported that oridonin alleviates early PF caused by lipopolysaccharide by inhibiting the NLRP3 inflammasome, NOX4-dependent oxidative imbalance, impaired autophagy, and EMT (Yang L. et al., 2022). Zhang et al. examined the combined effects of Schizandrin B (Sch B) and Glycyrrhizic acid (GA) on BLM-induced PF. The combination inhibits the TGF-β1/Smad2 signaling pathway and overexpression of NOX4 (Zhang et al., 2017). These studies underscore the potential of TCM as multi-modal therapeutics for PF, offering advantages in targeting interconnected pathological pathways with minimal side effects.

During PF progression, there is a close relationship between the TGF-β1/Smad signaling pathway and ROS generation (Jia et al., 2019). TGF-β1 can promote the formation of ROS by inducing the expression of ROS-producing enzyme NOX4 (Cui et al., 2021). Andrographolide has been shown to mitigate TGF-β1-induced EMT in alveolar epithelial A549 cells by suppressing both Smad2/3 and Erk1/2 signaling pathways activated by TGF-β1, alongside significantly reducing ROS levels in the process (Li et al., 2020). Another study found that Bruceine A exerts antifibrotic effects by targeting galectin-3 to disrupt its interaction with TGF-β1, thereby inhibiting Smad-dependent signaling pathways and slowing fibrotic progression (Du et al., 2025). By targeting these interconnected pathways, such TCMs illustrate the potential for multifaceted approaches to fibrosis treatment. Their integration into modern medical practices could give rise to innovative, comprehensive strategies against PF.

### 3.3 Inhibition of PUFAs peroxidation

Lipids containing diallyl carbons and PUFAs are highly susceptible to lipid peroxidation, which can induce ferroptosis (Kagan et al., 2017). Ferroptosis is pivotal in the development of PF. The metabolism of PUFAs also changes during the development of PF. AA, a type of PUFAs, is crucial in ferroptosis and serves as a precursor to inflammatory mediators, which can be converted into various substances that exacerbate inflammation and promote the development of fibrosis (Charbeneau and Peters-Golden, 2005). Gao et al. found that a low dose of Qingwen Gupi Decoction (QGT) alleviates inflammation and fibrotic tissue damage in a rat fibrosis model. Metabolomic analysis revealed that QGT’s anti-fibrotic effects are linked to changes in AA metabolism. QGT can regulate key metabolic biomarkers of AA, such as increasing 15-hydroxyeicosatetraenoic acid levels and downregulating TGF-β1 and Smad3 expression, thereby hindering PF progression (Gao et al., 2023). Moreover, Chen et al. analyzed the Shuangshen Pingfei Formula (SSPF) used for PF treatment and identified the involvement of the AA metabolic pathway in PF (Chen T. et al., 2024). Both QGT and SSPF, as TCM compounds, demonstrate therapeutic efficacy against PF by modulating PUFA metabolism, particularly targeting AA-related pathways. These multi-component formulations showcase the advantages of TCM in addressing inflammation, oxidative stress, and ECM remodeling through multi-pathway regulation. Their holistic action, supported by metabolomic and transcriptomic evidence, highlights the potential of integrating multi-omics approaches to unravel synergistic mechanisms and develop new strategies for treating PF with reduced systemic toxicity.

ACSL4, a key gene in ferroptosis, can facilitate this process, leading to iron overload and enhanced lipid peroxidation within cells, which, in turn, causes cellular damage and death. These changes can further initiate an inflammatory response, activate fibroblasts, and prompt them to secrete a large amount of ECM such as collagen, ultimately resulting in PF. Research by Wen et al. demonstrated that a decoction of Astragalus and Panax notoginseng can reduce ROS levels in lung tissues, downregulate ACSL4 expression, inhibit ferroptosis, and thus alleviate PF (Wen et al., 2024). In a BLM-induced PF mouse model, lipoxygenase 2 (LOX2) expression is upregulated in lung tissues. Tao et al. discovered that piceid can suppress both the expression and activity of LOX2. *In vitro* experiments demonstrated that treating primary mouse lung fibroblasts stimulated by TGF-β1 with piceid can reduce LOX2 expression. *In vivo* experiments also showed that piceid can counteract weight loss in BLM-induced mice, increase survival rates, and decrease the lung index. Additionally, in the lung tissues of PF mice treated with piceid, both the expression and mRNA levels of LOX2 are decreased, thereby alleviating PF (Tao et al., 2017). Therefore, investigating the relationships among PUFAs, ferroptosis, and PF is crucial for advancing our understanding of PF pathogenesis and identifying potential new therapeutic targets.

### 3.4 Regulation of antioxidant system

The GSH/GPX4 system is recognized as a major factor in preventing peroxidative damage and thus decelerating the progression of ferroptosis, which plays a vital role in the pathogenesis of PF. By maintaining adequate GSH levels and GPX4 functionality, along with the efficient functioning of the system Xc^−^, the antioxidant defense mechanism is strengthened, thereby suppressing ferroptosis and alleviating PF (Zhang T. et al., 2024). Shariati et al. investigated the protective effects of epicatechin (Epi), a flavonoid known for its antioxidant and anti-inflammatory properties, against BLM-induced pulmonary oxidative stress, inflammation, and fibrosis in mice. The animals were divided into groups and received different doses of Epi before and after BLM administration. The findings revealed that Epi markedly reduces oxidative stress markers (e.g., increased activities of SOD, CAT, GPX, and GSH levels, while decreasing MDA levels) and fibrotic markers (e.g., reduced hydroxyproline and TGF-β levels) in a dose-dependent manner. Histopathological analysis corroborated that Epi alleviates alveolitis, inflammation, and collagen deposition. The protective mechanism of Epi is attributed to its antioxidant properties, including free radical scavenging and metal ion chelation, which mitigates BLM-induced lung injury (Shariati et al., 2019). Similarly, Mehrabani et al. demonstrated that crocin administration significantly decreases tumor necrosis factor alpha (TNF-α), MDA, and nitric oxide (NO) levels in BLM-induced PF models. This therapeutic approach also bolsters pulmonary antioxidant defenses, as evidenced by increased enzymatic activities of GSH, CAT, and GPX (Mehrabani et al., 2020). In another study, Peimine relieves BLM-induced PF by upregulating E-cadherin and downregulating vimentin, while also reducing the expression of interleukin-1β (IL-1β), interleukin-6 (IL-6), and TNF-α, increasing SOD and GPX activities, and lowering MDA levels in the lungs (Li L. et al., 2023). In summary, the TCM ingredients mentioned above, along with others listed in Table 1, illustrate the benefits of TCM-derived interventions in PF treatment: multi-pathway regulation, ferroptosis inhibition through antioxidant reinforcement, and minimized toxicity. These properties make TCM promising candidates for integrative strategies against PF, combining traditional knowledge with modern mechanistic insights.

**TABLE 1:** Potential therapeutic drugs targeting ferroptosis for PF.

Classification	Therapeutic agents	Targeting molecule	Potential mechanism	Application	References
Regulation of iron metabolism	Dihydroquercetin	Ferritinophagy inhibitor	Decrease the accumulation of iron and lipid peroxidation products, and increase levels of GSH and GPX4	Alleviate PF	Yuan et al. (2022)
	Fraxetin	NCOA4 inhibitor	Inhibit NCOA4	Alleviate PF	Zhai et al. (2023)
	Qingfei Xieding prescription	ACE2-ERK agonist	Activate ACE2-ERK signaling axis, reduce the levels of MDA, ROS and Fe^2+^	Alleviate PF	Sun et al. (2024a)
Inhibition of ROS generation	Shenks	NOX4 inhibitor	Block TGF-β pathway and inhibit NOX4 and ROS	Inhibit PF	Chu et al. (2017)
	Oridonin	NOX4 inhibitor	Inhibit NOX-4-dependent oxidative imbalance and induce the overexpression of Nrf2 and HO-1	Alleviate early PF	Yang et al. (2022a)
	Schizandrin B and Glycyrrhizic acid	NOX4 inhibitor	Inhibit TGF-β1/Smad2 signaling pathway and overexpression of NOX4	Synergistic protection against PF	Zhang et al. (2017)
	Arenaria kansuensis	NOX4 inhibitor	Downregulate NOX4, activate of Nrf2 pathway and inhibit NF-κB/TGF-β1/Smad2/3 pathway	Attenuate PF	Cui et al. (2021)
	Jinshui Huanxian formula	NOX4 inhibitor	Restore the balance of Nrf2–NOX4	Treat PF	Bai et al. (2018)
	Gallic acid derivative	NOX4 inhibitor	Inhibit TGF-β1/Smad2 signaling pathway and balance NOX4/Nrf2	Alleviate PF	Rong et al. (2018)
	Qing Fei Hua Xian Decoction	NOX4 inhibitor	Restore the balance of Nrf2–NOX4, ACE-AngII-AT1R and ACE2-Ang-(1–7)-Mas axes	Treat PF	Li et al. (2023b)
	Salvia miltiorrhiza	NOX4 inhibitor	Reduce PKC-δ/Smad3 signaling activation and balance NOX4/Nrf2	Alleviate PF	Peng et al. (2019)
	Costunolide	NOX4 inhibitor	Block NF-κB and TGF-β1/Smad2/Nrf2-NOX4 signaling pathways	Inhibit PF	Liu et al. (2019)
	Mogrol	NOX4 inhibitor	Activate AMPK and ameliorate TGF-β1 signaling pathway, restore NOX4 abnormal expression	Against PF	Liu et al. (2021)
	Ginkgetin	NOX4 inhibitor	Promote AMPK phosphorylation and Sirt1 expression, inhibit NOX4	Against PF	Ren et al. (2023)
	Tanshinone IIA	NOX4 inhibitor	Reduce PKC-δ/Smad3 signaling activation and balance NOX4/Nrf2/GSH; suppress TGF-β1/Smad signaling, inhibit NOX4 expression and activate the Nrf2/ARE pathway	Restrain PF; attenuate silica-induced PF	An et al. (2019), Feng et al. (2019)
	*Lonicera japonica*	NF-κB/TGF-β1 inhibitor	Inhibit TGF-β and NF-κB signaling pathways, regulate the production of ROS	Against PM2.5 induced PF	Lee et al. (2023)
	Quercetin-3-Rutinoside	NF-κB/TGF-β1 inhibitor	Inhibit NF-κB/TGF-β1 signaling, decrease ROS and NO	Alleviate radiation-induced PF	Verma et al. (2022)
	Mangiferin	TGF-β1/Smad inhibitor	Inhibit TLR4/p65 and TGF-β1/Smad2/3 pathway, downregulate ROS generation	Attenuate PF	Jia et al. (2019)
	Andrographolide	TGF-β1/Smad inhibitor	Suppress TGF-β1/Smad2/3/Erk1/2 signaling pathways, reduce ROS	Treat PF	Li et al. (2020)
	Bruceine A	TGF-β1/Smad inhibitor	Inhibit TGF-β1/Smad pathway, reduce MMP and ROS	Prevent PF	Du et al. (2025)
Inhibition of PUFAs peroxidation	Qingwen Gupi decoction	TGF-β1/Smad inhibitor	Downregulate TGF-β1 and Smad-3, intervene AA, GPL and Phe metabolism	Alleviate PF	Gao et al. (2023)
	Shuangshen Pingfei formula	AA metabolic pathway regulator	Regulate PF-related AA pathway metabolites	Alleviate PF	Chen et al. (2024b)
	Astragalus and Panax notoginseng decoction	Hif-1α-EGFR agonist	Activate Hif-1α-EGFR signaling pathway, downregulate ACSL4, PTGS2, and ROS while upregulating GPX4 and SLC7A11	Alleviate PF	Wen et al. (2024)
	Rhapontin	AMPK agonist	Activate AMPK and suppress the TGF-β/Smad pathway, decrease LOX2 and p-Smad2/3	Prevent PF	Tao et al. (2017)
Regulation of antioxidant system	Epicatechin	Antioxidant	Increase the activity of GPX, SOD, CAT and GSH level, reduce tissue levels of MDA, HP, TGF-β and lung index	Against pulmonary oxidative stress, inflammation and fibrosis	Shariati et al. (2019)
	Crocin	Antioxidant; Nrf2/HO-1 agonist	Decrease TNF-α, MDA and NO levels, increase GSH, CAT and GPX; activate Nrf2/HO-1 signal pathway	Against PF	Zaghloul et al. (2019), Mehrabani et al. (2020)
	Peimine	Antioxidant	Decrease HYP, vimentin, IL-1β, IL-6 and TNF-α, increase SOD and GPX	Ameliorate PF	Li et al. (2023c)
	Chuanxiong Kangxian granules	Antioxidant	Reduce the levels of SOD and GSH	Ameliorate PF	Shi et al. (2017)
	Polydatin	Antioxidant	Restore GSH content and hinder MDA and 8-OHdG levels	Against PF	Ali et al. (2022)
	Andrographolide	Antioxidant	Reduce NIT, MDA and upregulate GSH	Against silica induced PF	Karkale et al. (2018)
	Pachymic Acid	Antioxidant	Reduce IL-6 and TNF-α, increase IL-10. Decrease HYP and MDA, and increase SOD and GPX. Inhibit NLRP3, ASC, IL-1β, P20, and TXNIP	Against PF	Wang et al. (2023)
	Chrysin	Antioxidant	Reduce HYP, TGF-β1, TXNIP, MDA, NOx, iNOS, HIF-1α and increase GSH and SOD	Mitigate PF	Kseibati et al. (2020)
	Rutin	Antioxidant	Decrease HYP, MDA, NO, TGF-β1, α-SMA/Col I and III, increase SOD and GSH	Ameliorate PF	Bai et al. (2020)
	Chlorogenic acid	Antioxidant	Increase GPX, CAT and SOD	Prevent oxidative and fibrotic injuries induced by PQ	Larki-Harchegani et al. (2023)
	Baicalin	Antioxidant	Decrease HYP and MDA, increase SOD, GSH-PX, T-SOD and GSH	Treat PF; suppress PF and fibroblast proliferation	Zhao et al. (2020), Chang et al. (2021)
	Quercetin and gallic acid	Antioxidant	Enhance SOD, GSH and decrease NO, IL-6	Attenuate PF	Mehrzadi et al. (2021)
	Schisandrin B	Antioxidant	Decrease HYP and TGF-β1, increase SOD and T-AOC	Attenuate PF	Wang et al. (2020)
	Hyperoside	Antioxidant	Reduce the levels of MDA, TNF-α, and IL-6 and increase the activity of SOD	Attenuate PF	Huang et al. (2020)
	Hesperetin	Antioxidant	Decrease IL-1β、IL-4、TNF-α, HYP and MDA, increase SOD, CAT, GPX, IFN-γ and IL-10	Attenuate silica-induced lung injury	Li et al. (2021a)
	Piceatannol	Antioxidant	Downregulate MDA, HDAC2, HDAC4 and TGF-β, increase GSH	Treat PF	Eldeen et al. (2023)
	Cinnamaldehyde	Antioxidant	Reduce HYP level and inhibit ROS production as well as enhance SOD	Ameliorate PF	Yan et al. (2018)
	Pachymic acid	Antioxidant	Increase the levels of SOD, CAT and ATP, decrease the activities of MDA and ROS	Treat PF	Li et al. (2023a)
	Tuberostemonine	SLC7A11/GSH transporter agonist	Enhance the expression of the SLC7A11/GSH transporter	Alleviate PF	Liu et al. (2024)
	Dihydroartemisinin	Nrf2/HO-1 agonist	Activate Nrf2/HO-1 signal pathway	Alleviate PF; mitigate RILI	Yang et al. (2018), Ning et al. (2024)
	Nobiletin	Nrf2/HO-1 agonist	Block PI3K/AKT/mTOR signaling, improve Nrf2/HO-1 pathway	Ameliorate PF	El Tabaa et al. (2023)
	Phillygenin	Nrf2/HO-1 agonist	Upregulate Nrf2, HO-1 and NQO-1, inhibit PI3K-Akt–mTOR signaling pathway	Prevent PF	Wei et al. (2025)
	Atractylenolide III	Nrf2 agonist	Activate Nrf2/NQO1/HO-1 pathway, increase SOD and GSH, decrease MDA and LDH	Attenuate PF	Huai and Ding (2020)
	Liquiritigenin	SIRT1/Nrf2 agonist	Activate SIRT1/Nrf2 signaling pathway	Against PF	Hua and Ren (2024)
	Zerumbone	SIRT1/Nrf2 agonist	Activate the SIRT1/Nrf2 pathway, enhance SOD, GSH-PX and decrease MDA, ROS	Alleviate PF	Bian et al. (2024b)
	Quercetin	Nrf2 agonist	Induce Nrf2	Anti-fibrogenic and anti-inflammatory effects; against PF	Veith et al. (2017), Boots et al. (2020)
	Tanshinone IIA	Nrf2 agonist	Activate Sesn2-Nrf2 signaling pathway; inhibit TGF-β1/Smad signaling pathway and activate Nrf2 signaling pathway	Restrain PF; attenuate silica-induced PF	Feng et al. (2020), Li et al. (2022)
	Protodioscin	Nrf2 agonist	Activate NBR1-p62-Nrf2 pathway	Inhibit PF	Zeng et al. (2024)
	Chelerythrine	Nrf2/ARE agonist	Activate the Nrf2/ARE pathway, upregulate SOD and GSH, decrease 4-HNE and HP, and increase HO-1 and NQO1	Manage PF	Peng et al. (2021)
	Emodin	Nrf2 agonist, TGF-β1 inhibitor	Suppress the TGF-β1/p-Smad-2/p-Smad-3, activate Nrf2 signaling process	Attenuate PF	Tian et al. (2018)
	Ephedrine	Nrf2 agonist, NF-κB inhibitor	Block NF-κB signaling and activate Nrf-2 signaling	Alleviate PF	Tian et al. (2023)
	Kurarinone	Nrf2/HO-1 agonist, TGF-β1 inhibitor	Suppress TGF-β signaling pathway, phosphorylation of Smad2/3 and AKT, activate Nrf2/HO-1 signal pathway	Attenuate PF	Park et al. (2021)
	Rosavin	Nrf2 agonist, NF-κB inhibitor	Downregulate HYP, MDA and increase SOD, GPX. Increase Nrf2, inhibit NF-κB p65, TGF-β1 and α-SMA.	Treat PF	Xin et al. (2019)
	Salvianolic acid B	Nrf2 agonist; MAPK and NF-κB inhibitor	Reduce ROS and MDA, increase GSH, induce Nrf2 activation; inhibit MAPK and NF-κB signaling pathways, against oxidative stress injury	Treat PF; attenuate PF	Liu et al. (2018a), Liu et al. (2018b)
	Sinapic acid	Nrf2/HO-1 agonist, NF-κB inhibitor	Downregulate NF-κB, and activate Nrf2/HO-1 signal pathway	Ameliorate PF	Raish et al. (2018)
	Modified Qing-Zao-Jiu-Fei decoction	NF-κB/Nrf2 and MAPK inhibitor	Suppress the activation of NF-κB/Nrf2 and MAPKs pathways, increase GSH-PX, SOD, GSH and decrease MDA	Treat PF	Zhu et al. (2024)
	Hesperidin	TGF-β1/Smad3/AMPK and NF-κB inhibitor, Nrf2/HO-1 agonist	Inhibit TGF-β1/Smad3/AMPK and IκBα/NF-κB pathways, inhibit downregulated Nrf2 and HO-1 as well as upregulated TNF-α, IL-1β, IL-6, collagen-1, TGF-β, and Smad-3 mRNA	Ameliorate PF	Zhou et al. (2019)
	Anemarrhenae Rhizoma	TGF-β1/Smad inhibitor	Inhibit TGF-β1/Smad signaling pathway, decrease MPO and NO, enhance enzymatic antioxidants activity	Alleviate PF	Shen et al. (2022)
	Resveratrol	TGF-β1/Smad inhibitor	Downregulate the TLR4/NF-κB and TGF-β1/smad3 signaling pathways, increase SOD and GPX	Alleviate PF	Wang et al. (2022)
	Polydatin	TGF-β1 inhibitor	Inhibit TGF-β1 expression and phosphorylation of Smad 2 and 3 and ERK-1 and -2, reduce the levels of HYP, TNF-α, IL-6, IL-13, MPO and MDA and promote SOD activity	Prevent PF	Liu et al. (2020)
	Zingerone	TGF-β1 and iNOS inhibitor	Decrease TNF-α, IL-1β, MDA, TGF-β1, and iNOS and increase SOD and GPX	Against PF	Gungor et al. (2020)
	Wogonin	MAPK inhibitor	Suppress the MAPK pathway, increase GSH-PX, SOD and decrease MDA	Manage PF	Bian et al. (2024a)

As the key transcriptional regulator of cytoprotective responses, Nrf2 coordinates the expression of antioxidant genes crucial for cellular redox homeostasis. Nrf2 inhibits ferroptosis by suppressing lipid peroxidation cascades through downstream effectors like heme oxygenase-1 (HO-1) and SLC7A11 (Xu et al., 2024). Yang et al. studied dihydroartemisinin (DHA) in rats with BLM-induced PF and discovered that DHA significantly lowers oxidative stress (e.g., decreased MDA levels, increased SOD and GSH activities), reduces collagen synthesis, and prevents alveolar epithelial cells from differentiating into myofibroblasts. These positive effects are associated with the upregulation of the Nrf2/HO-1 signaling pathway, as evidenced by increased Nrf2 and HO-1 protein and mRNA expressions in lung tissues. Animal experiments further demonstrated that DHA-treated rats have lower alveolitis severity and fibrosis scores compared to control groups. These findings suggest that DHA could be a potential treatment for PF by modulating the Nrf2/HO-1 signaling pathway to alleviate oxidative stress (Yang et al., 2018). Moreover, Nrf2 interacts with signaling pathways such as TGF-β1, NF-κB, and MAPK, which are crucial in oxidative stress-related pathologies and chronic inflammation (Tian et al., 2018; Zhu et al., 2024). Feng et al. showed that Tanshinone IIA (Tan IIA) mitigates silica-induced oxidative damage by enhancing Nrf2-dependent antioxidant defenses, significantly inhibiting fibrotic matrix deposition in experimental silicosis through the modulation of EMT dynamics and TGF-β1/Smad3 transduction cascades (Feng et al., 2020). In another study, Salvianolic acid B improves inflammatory responses in PF by maintaining endothelial cell integrity under oxidative stress, suppressing vascular hyperpermeability and pro-inflammatory cytokine production through dual modulation of MAPK/NF-κB transduction (Liu M. et al., 2018). These findings underscore the unique advantages of herbal medicine in treating PF through multi-target modulation of oxidative stress and fibrotic signaling cascades. This multi-target approach aligns with TCM principles, offering a holistic treatment method with fewer off-target effects compared to single-pathway inhibitors. The ability of these phytochemicals to maintain cellular redox homeostasis, inhibit EMT, and reduce ECM deposition highlights their potential as safer, natural alternatives or supplements to conventional antifibrotic therapies. Combining TCM’s empirical wisdom with modern mechanistic insights could lead to innovative, precision treatments for oxidative stress-related fibrotic diseases.

## 4 Integrating TCM with nanotechnology to inhibit ferroptosis in PF

Notably, the chronic systemic administration of conventional anti-fibrotic therapies often leads to issues such as low solubility, poor stability, rapid clearance, and dose-limiting side effects (Wan et al., 2023). In this regard, nanoscale drug carriers present significant benefits for optimizing physicochemical properties and absorption kinetics. These engineered systems enhance drug solubility and biodistribution parameters while facilitating targeted cellular internalization. Specifically, nanoparticle formulations designed for pulmonary use show better alveolar deposition efficiency and extended tissue residence time through controlled release mechanisms. These technological innovations enable dose reduction while maintaining therapeutic efficacy, thereby achieving optimized pharmacokinetic profiles with reduced systemic toxicity (Pramanik et al., 2021; Loo and Lee, 2022).

### 4.1 ROS-scavenging TCM nanoplatforms mitigate PF

**FIGURE 2:**
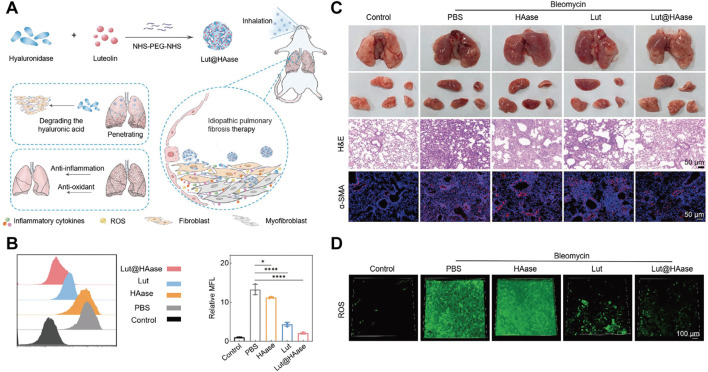
**(A)** Schematic illustration of Lut@HAase with efficient anti-fibrotic performance for the treatment of PF. **(B)** The flow cytometry histogram and quantitative analysis of potential ROS clearance from different treatments for fibrotic cells (n = 3). **(C)** Representative images of lungs from mice treated with PBS, HAase, Lut, or Lut@HAase after BLM challenge, H&E staining and immunofluorescence staining of α-SMA in lung sections (Scale bars: 50 μm). **(D)** The levels of ROS in lung tissues from different treatment groups (green: ROS; scale bar: 100 μm). Reprinted with permission from (Pan et al., 2024). Copyright (2024) Wiley-VCH.

PF is a progressive lung disease induced by oxidative stress and is closely linked to ROS-induced cellular damage and ferroptosis. Targeted delivery of ROS-scavenging TCM through nanocarriers demonstrates great potential in inhibiting ferroptosis thus exerting superior PF treatment effect. In one study, Pan et al. developed a nanopreparation containing Luteolin’s hyaluronidase nanoparticles (referred to as Lut@HAase), which can be locally accumulated in the lungs through non-invasive inhalation to treat PF, thereby enhancing Lut penetration into lesions and promoting ROS scavenging (Figure 2A). *In vitro* studies on TGF-β1-stimulated MRC5 fibroblasts demonstrated that Lut@HAase achieves a 70% reduction in mean fluorescence intensity of ROS, compared to a 40% reduction with free Lut (Figure 2B). *In vivo* experiments indicated that Lut@HAase significantly reduces lung tissue damage, as shown by histological examination. Hematoxylin and eosin (H&E) staining showed that lung interstitial cells return to an elongated and flat morphology after Lut@HAase treatment. Moreover, immunofluorescence staining demonstrated decreased α-SMA expression after Lut@HAase treatment, consistent with its antifibrotic action (Figure 2C). In contrast to BLM-induced mice exposed to HAase or Lut alone, those treated with Lut@HAase has ROS levels closest to the control group (Figure 2D) (Pan et al., 2024). In another study, Zheng et al. designed a nanoplatform (AS_LIG@PPGC NPs) co-encapsulating astragaloside IV (AS) and ligustrazine (LIG), which demonstrated potent anti-fibrotic efficacy through dual-pathway suppression of NOX4-mediated ROS/p38 MAPK and NLRP3 signaling, reducing ROS generation while disrupting the self-amplifying loop between NOX4 activation and inflammasome formation (Zheng et al., 2024). Additionally, Yao et al. developed a quercetin delivery system encapsulated by chitosan-based nanoparticles (Qu/CS-NPs) through ionic interaction, improving water solubility and stability of quercetin. This system significantly alleviates silica-induced PF by reducing oxidative stress, inhibiting inflammatory factor release, and decreasing collagen accumulation (Yao et al., 2023).

### 4.2 Antioxidant system-regulating TCM nanoplatforms mitigate PF

Nanoplatforms have shown great potential in modulating antioxidant systems, offering new methods to address PF. In one study, Zhang et al. developed taraxasterol (TA) loaded methoxy poly (ethylene glycol)-poly (D, L-lactide) (mPEG-PLA) and D-α-tocopheryl polyethylene glycol succinate (TPGS) mixed polymeric micelles (TA-PM) to enhance the properties of TA for pulmonary applications. In the *in vitro* study, TA inhibits EMT by downregulating mesenchymal markers vimentin and α-SMA, and upregulating the epithelial marker E-cadherin in A549 cells treated with TGF-β1. In a BLM-induced PF mouse model, TA-PM treatment reduces lung inflammation, oxidative stress, and fibrosis. H&E staining revealed that TA-PM treatment normalizes alveolar structure and decreases inflammation. Masson staining indicated that TA-PM alleviates collagen deposition. Immunohistochemistry showed that TA-PM significantly reduces α-SMA expression. Additionally, TA-PM reverses BLM-induced oxidative stress, with high-dose TA-PM almost normalizing GSH, SOD, and MDA levels (Zhang F. et al., 2024). Khawas et al. developed and evaluated umbelliferone (UMB)-loaded nanostructured lipid carriers (NLCs) for PF treatment. UMB-NLC mitigates PF by reducing oxidative stress through suppression of lipid peroxidation (MDA) and restoration of antioxidant defenses (like GSH, SOD, and CAT), counteracting fibrosis-promoting pathways (Khawas et al., 2024). In another study, Sherekar et al. prepared polylactic-co-glycolic acid (PLGA) nanoparticles loaded with diosgenin (DG) and emodin (ED). The findings showed that the designed nanoplatforms can reduce MDA, NADPH, and protein carbonyl levels, while enhancing GSH, SOD, and CAT activities. They also downregulate the expression of TNF-α, IL-1β, IL-6, monocyte chemotactic protein 1, and TGF-β1, effectively alleviating PF (Sherekar et al., 2024). These cases showcase the innovation in nanoplatform design and anti-fibrotic mechanism exploration. However, more progress is needed in understanding mechanisms, ensuring long-term safety, and optimizing clinical translation (such as dosage form optimization and comparative trials) in order to advance from laboratory research to practical applications.

### 4.3 Application of advanced biomedical engineering technologies in PF prevention, diagnosis, and treatment

**FIGURE 3:**
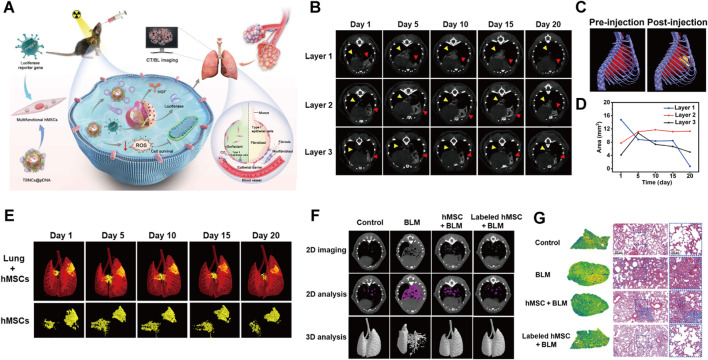
**(A)** Schematic depicting TBNCs@pDNA-mediated gene delivery and dual-modal imaging to track hMSCs in PF therapy. **(B)** *In vivo* micro-CT images of labeled hMSCs in PF mouse lungs at days 1, 5, 10, 15, and 20 post-transplantation. **(C)** Pre- and post-transplantation 3D *in vivo* micro-CT of labeled hMSCs in PF mice. **(D)** Graph of labeled hMSC CT signal distribution versus post-transplantation time. **(E)** 3D CT images of labeled hMSCs in PF mouse lungs at days 1, 5, 10, 15, and 20 post-transplantation. **(F)** Micro-CT images of Control, BLM, and BLM mice treated with hMSCs or labeled hMSCs. **(G)** Masson’s trichrome staining of lung tissues from Control, BLM, and BLM mice treated with hMSCs or labeled hMSCs. Reprinted with permission from (Bao et al., 2024). Copyright (2024) American Association for the Advancement of Science.

The latest progress in PF research is driven by the collaborative integration of state-of-the-art technologies. Mesenchymal stem cells (MSCs) offer significant potential for treating PF due to their established safety profile and remarkable paracrine effects. Bao et al. developed AuPtCoPS trimetallic-based nanocarriers (TBNCs) with enzyme-like activity and DNA loading. These TBNCs combat oxidative stress, deliver therapeutic genes, and enable CT tracking of human MSCs (hMSCs) (Figure 3A). Following transplantation into PF mice, hMSCs were identified in the lungs (Figure 3C). CT imaging revealed a decrease in the signal area indicated by the yellow arrow in layer 1 over time; however, CT values increased on days 5 and 10, likely reflecting hMSCs migration to fibrotic sites. Concurrently, the signal represented by the red arrow in layer 1 significantly diminished by day 5, while signals in layers 2 and 3 exhibited an increase, suggesting downward migration of hMSCs (Figures 3B,D). 3D CT images confirmed hMSCs proliferation on day 5 followed by a subsequent reduction in signal intensity (Figure 3E **)**. This approach facilitated real-time observation of hMSC distribution, migration patterns, and biological activities, thereby visualizing their therapeutic efficacy and assisting in optimizing hMSCs-based therapies. Additionally, both 2D and 3D CT demonstrated improved lung ventilation in the labeled hMSCs-treated group compared to the BLM group (Figure 3F). Histopathological examination revealed reduced collagen deposition and scar formation within the labeled hMSCs group relative to the BLM group (Figure 3G). Overall, this study presents an efficient and promising MSCs therapy for PF (Bao et al., 2024). In addition, CRISPR-Cas9 gene editing enables precise modification of PF-related genes such as desmoplakin (DSP) and CD5 molecule-like (CD5L), presenting new strategies to reduce genetic risk and develop targeted therapies (Qu et al., 2018; Guo et al., 2023). Moreover, high-throughput multi-omics platforms have unveiled the molecular mechanisms underlying therapeutic agents; Ge et al. demonstrated that demethyleneberberine suppresses gremlin-1 stability by blocking ubiquitin-specific protease 11 (USP11)-mediated deubiquitination, revealing its critical binding interface through proteomic profiling (Ge et al., 2024). Advanced three-dimensional (3D) models have become essential tools for understanding PF mechanisms by accurately simulating its pathological microenvironments. These models successfully replicate key features such as abnormal ECM deposition, matrix stiffness gradients, and dynamic EMT (Jain et al., 2024). In terms of diagnostic innovations, artificial intelligence (AI)-enhanced analysis of high-resolution computed tomography imaging patterns, as validated by Chantzi et al., greatly improves the accuracy of PF classification and prognostic stratification (Chantzi et al., 2025). Additionally, liquid biopsy technologies that detect disease-specific circulating free cell DNA (cfDNA) signatures are being developed, with Pallante’s research establishing plasma cfDNA as a distinctive biomarker for PF compared to other interstitial lung diseases (Pallante et al., 2021). Together, these integrated technologies—including gene editing, mechanism-based drug discovery, AI-powered imaging analytics, and minimally invasive diagnostics—create a multidimensional framework to advance precision medicine in PF management.

## 5 Conclusion, challenges and outlook

This review highlights the pivotal role of ferroptosis in PF and emphasizes the importance of identifying ferroptosis-related therapeutic targets, such as iron metabolism, ROS production, PUFAs metabolism, and the antioxidant system, as key pathways for therapeutic intervention. TCM has shown promise in modulating ferroptosis through its multi-component, multi-target, and multi-pathway advantages. Studies have demonstrated that TCM formulations, extracts, and monomers can effectively regulate iron metabolism, reduce ROS production, inhibit PUFAs peroxidation, and restore antioxidant defenses, thereby alleviating PF. Furthermore, the integration of nanotechnology with TCM enhances therapeutic outcomes by improving drug bioavailability and enabling precise targeting of lung pathology, thus offering innovative treatments that address oxidative stress and inflammation in PF.

Despite the promising potential of TCM in preventing and treating PF, several limitations must be addressed. Firstly, the precise molecular mechanisms through which TCM, whether in combined formulas or single-agent forms, regulates iron metabolism or oxidative stress are still unclear, which complicates its integration into modern precision medicine. Secondly, most research on ferroptosis has been conducted using preclinical models, and there is a lack of clinical trials with patients and biomarkers linked to ferroptosis. Consequently, it will take considerable time before these TCMs to be applied clinically. Thirdly, although nanotechnology can enhance TCM delivery, developing safe and effective nanocarriers requires rigorous testing to prevent potential toxicity and immune responses. Moreover, the heterogeneity of PF poses a significant challenge. PF can arise from various causes, including environmental exposures, genetic predispositions, and autoimmune diseases. This heterogeneity complicates the development of universal therapeutic strategies, as different PF subtypes may require customized approaches.

Looking forward, the integration of TCM with contemporary biomedical technologies holds great promise for the treatment of PF. Progress in omics technologies, such as genomics, proteomics, and metabolomics, can unveil intricate details about the molecular workings of TCM and its impact on ferroptosis. These advancements also facilitate the discovery of new biomarkers for PF, paving the way for earlier diagnosis and more precise therapeutic targeting. The innovation of new nanocarriers for TCM delivery presents another exciting prospect. Future research should aim to create nanocarriers capable of selectively targeting fibrotic lung tissue while minimizing unintended effects. Additionally, the combination of TCM with existing pharmacological treatments like pirfenidone and nintedanib could offer synergistic benefits, potentially reducing required doses and side effects. Furthermore, investigating ferroptosis in other fibrotic diseases, such as liver and cardiac fibrosis, could broaden the therapeutic use of TCM. The common mechanisms of ferroptosis across various fibrotic conditions suggest that TCM compounds targeting this process might possess immense therapeutic potential.

To conclude, despite the considerable advancements in comprehending the role of ferroptosis in PF and the potential of TCM to influence this process, a few hurdles still exist. Tackling these issues through cross-disciplinary research and cutting-edge technologies is essential for converting these insights into practical treatments for PF and other fibrotic conditions. The future of PF therapy hinges on integrating traditional wisdom with contemporary scientific approaches, offering hope for patients suffering from this debilitating disease.

## Glossary

PF: pulmonary fibrosis
TCM: traditional Chinese medicine
ROS: reactive oxygen species
PUFAs: polyunsaturated fatty acids
GSH: glutathione
GPX4: glutathione peroxidase 4
BLM: bleomycin
AECIIs: alveolar epithelial type II cells
NADPH: nicotinamide adenine dinucleotide phosphate
NOXs: NADPH oxidases
XO: xanthine oxidase
NOS: nitric oxide synthase
TGF-β1: transforming growth factor-β1
NOX4: NADPH oxidase 4
NLRP3: NOD-like receptor thermal protein domain-associated protein 3
AA: arachidonic acid
ACSL4: acyl-CoA synthetase long chain member 4
LPCAT3: lysophosphatidylcholine acyltransferase 3
LOXs: lipoxygenases
PUFA-OOH: polyunsaturated fatty acid hydroperoxide
IL-4: interleukin-4
IL-13: interleukin-13
CAT: catalase
SOD: superoxide dismutase
MDA: malondialdehyde
SLC7A11: solute carrier family 7 member 11
MAPKs: smitogen-activated protein kinases
ERK: extracellular signal-regulated kinase
JNK: c-Jun N-terminal kinase
NF-κB: nuclear factor kappa-B
Nrf2: nuclear factor erythroid 2-related factor 2
NCOA4: nuclear receptor coactivator 4
LIP: labile iron pool
DHQ: dihydroquercetin
CTGF: connective tissue growth factor
LOX2: lipoxygenase 2
TNF-α: tumor necrosis factor alpha
NO: nitric oxide
IL-1β: interleukin-1β
IL-6: interleukin-6
HO-1: heme oxygenase-1
DHA: dihydroartemisinin
α-SMA: α-smooth muscle actin
H&E: hematoxylin and eosin
AECs: alveolar epithelial cells
MSCs: mesenchymal stem cells
hMSCs: human MSCs
DSP: desmoplakin
CD5L: CD5 molecule-like
USP11: ubiquitin-specific protease 11
3D: three-dimensional
AI: artificial intelligence
cfDNA: circulating free cell DNA
DAMPs: damage-associated molecular patterns
PDGF: platelet-derived growth factor
MMP-9: matrix metalloproteinase-9
EMT: epithelial-mesenchymal transformation
ECM: extracellular matrix

## References

[B1] Alessandro A. Anna Martina B. Alessandro S. Lavinia P. Emanuele G. Selene B. (2024). Ferroptosis and oral squamous cell carcinoma: connecting the dots to move forward. Front. Oral Health 5 (0), 1461022. 10.3389/froh.2024.1461022 39296524 PMC11408306

[B2] Ali M. K. Kim R. Y. Brown A. C. Donovan C. Vanka K. S. Mayall J. R. (2020). Critical role for iron accumulation in the pathogenesis of fibrotic lung disease. J. Pathology 251 (1), 49–62. 10.1002/path.5401 32083318

[B3] Ali Y. A. Ahmed A. A. E. Abd El-Raouf O. M. Elkhoely A. Gad A. M. (2022). Polydatin combats methotrexate-induced pulmonary fibrosis in rats: involvement of biochemical and histopathological assessment. J. Biochem. Mol. Toxicol. 36 (5), 9. 10.1002/jbt.23019 35174937

[B4] Amara N. Goven D. Prost F. Muloway R. Crestani B. Boczkowski J. (2010). NOX4/NADPH oxidase expression is increased in pulmonary fibroblasts from patients with idiopathic pulmonary fibrosis and mediates TGFbeta1-induced fibroblast differentiation into myofibroblasts. Thorax 65 (8), 733–738. 10.1136/thx.2009.113456 20685750 PMC3004009

[B5] An L. Peng L. Y. Sun N. Y. Yang Y. L. Zhang X. W. Li B. (2019). Tanshinone IIA activates nuclear factor-erythroid 2-Related factor 2 to restrain pulmonary fibrosis *via* regulation of redox homeostasis and glutaminolysis. Antioxidants and Redox Signal. 30 (15), 1831–1848. 10.1089/ars.2018.7569 30105924

[B6] Anna Martina B. Alessandro S. Eleonora V. Stefania S. Lavinia P. Emanuele G. (2023). Iron affects the sphere-forming ability of ovarian cancer cells in non-adherent culture conditions. Front. Cell Dev. Biol. 11 (0), 1272667. 10.3389/fcell.2023.1272667 38033861 PMC10682100

[B7] Bai L. L. Li A. M. Gong C. K. Ning X. C. Wang Z. H. (2020). Protective effect of rutin against bleomycin induced lung fibrosis: involvement of TGF-β1/α-SMA/Col I and III pathway. Biofactors 46 (4), 637–644. 10.1002/biof.1629 32233122

[B8] Bai Y. P. Li J. S. Zhao P. Li Y. Li M. Feng S. X. (2018). A Chinese herbal formula ameliorates pulmonary fibrosis by inhibiting oxidative stress *via* upregulating Nrf2. Front. Pharmacol. 9, 628. 10.3389/fphar.2018.00628 29946261 PMC6005894

[B9] Bao H. Y. Wu M. X. Xing J. Li Z. H. Zhang Y. N. Wu A. G. (2024). Enzyme-like nanoparticle-engineered mesenchymal stem cell secreting HGF promotes visualized therapy for idiopathic pulmonary fibrosis *in vivo* . Sci. Adv. 10 (34), eadq0703. 10.1126/sciadv.adq0703 39167646 PMC11338238

[B10] Bian B. Ge C. Wu F. W. Fan Y. L. Kong J. L. Li K. (2024a). Wogonin Attenuates bleomycin-induced pulmonary Fibrosis and oxidative stress injury *via* the MAPK signaling pathway. Biol. and Pharm. Bull. 47 (12), 2165–2172. 10.1248/bpb.b24-00534 39756931

[B11] Bian Y. L. Yin D. Q. Zhang P. Hong L. L. Yang M. (2024b). Zerumbone alleviated bleomycin-induced pulmonary fibrosis in mice *via* SIRT1/Nrf2 pathway. Naunyn-Schmiedebergs Archives Pharmacol. 397 (11), 8979–8992. 10.1007/s00210-024-03170-z 38874804

[B12] Boots A. W. Veith C. Albrecht C. Bartholome R. Drittij M. J. Claessen S. M. H. (2020). The dietary antioxidant Quercetin reduces hallmarks of bleomycin-induced lung fibrogenesis in mice. Bmc Pulm. Med. 20 (1), 112. 10.1186/s12890-020-1142-x 32349726 PMC7191795

[B13] Bruno G. Marcus C. Martina M. (2023). Mechanisms controlling cellular and systemic iron homeostasis. Nat. Rev. Mol. Cell Biol. 25 (2), 133–155. 10.1038/s41580-023-00648-1 37783783

[B14] Chang H. Meng H. Y. Bai W. F. Meng Q. G. (2021). A metabolomic approach to elucidate the inhibitory effects of baicalin in pulmonary fibrosis. Pharm. Biol. 59 (1), 1016–1025. 10.1080/13880209.2021.1950192 34362286 PMC8354164

[B15] Chantzi S. L. Kosvyra A. Chouvarda I. (2025). Radiomics and artificial intelligence in pulmonary fibrosis. J. Imaging Inf. Med. 14. 10.1007/s10278-024-01377-3 PMC12572475 39762544

[B16] Charbeneau R. P. Peters-Golden M. (2005). Eicosanoids: mediators and therapeutic targets in fibrotic lung disease. Clin. Sci. Lond. Engl. 108 (6), 479–491. 10.1042/cs20050012 15896193

[B17] Chen R. X. Dai J. H. (2023). Lipid metabolism in idiopathic pulmonary fibrosis: from pathogenesis to therapy. J. Mol. Medicine-Jmm 101 (8), 905–915. 10.1007/s00109-023-02336-1 37289208

[B18] Chen T. Ding L. Zhao M. R. Song S. Y. Hou J. Li X. Y. (2024a). Recent advances in the potential effects of natural products from traditional Chinese medicine against respiratory diseases targeting ferroptosis. Chin. Med. 19 (1), 49. 10.1186/s13020-024-00918-w 38519984 PMC10958864

[B19] Chen Y. Q. Liu J. Y. Sun Y. B. Li M. W. Fan X. S. Gu X. (2024b). Multi-omics study reveals shuangshen pingfei formula regulates EETs metabolic reprogramming to exert its therapeutic effect on pulmonary fibrosis. Int. Immunopharmacol. 143, 113275. 10.1016/j.intimp.2024.113275 39395378

[B20] Cheresh P. Kim S. J. Tulasiram S. Kamp D. W. (2013). Oxidative stress and pulmonary fibrosis. Biochimica Biophysica Acta-Molecular Basis Dis. 1832 (7), 1028–1040. 10.1016/j.bbadis.2012.11.021 PMC3639303 23219955

[B21] Chu H. Y. Shi Y. Jiang S. A. Zhong Q. C. Zhao Y. Q. Liu Q. M. (2017). Treatment effects of the traditional Chinese medicine shenks in bleomycin-induced lung fibrosis through regulation of TGF-beta/Smad3 signaling and oxidative stress. Sci. Rep. 7, 2252. 10.1038/s41598-017-02293-z 28533545 PMC5440393

[B22] Chung E. J. Reedy J. L. Kwon S. Patil S. Valle L. White A. O. (2019). 12-Lipoxygenase is a critical mediator of type II pneumocyte senescence, macrophage polarization and pulmonary fibrosis after irradiation. Radiat. Res. 192 (4), 367–379. 10.1667/rr15356.1 31373871 PMC6816027

[B23] Cui Y. L. Xin H. W. Tao Y. D. Mei L. J. Wang Z. (2021). Arenaria kansuensis attenuates pulmonary fibrosis in mice *via* the activation of Nrf2 pathway and the inhibition ofNF-kB/TGF-beta1/Smad2/3 pathway. Phytotherapy Res. 35 (2), 974–986. 10.1002/ptr.6857 32996197

[B24] Dixon S. J. Lemberg K. M. Lamprecht M. R. Skouta R. Zaitsev E. M. Gleason C. E. (2012). Ferroptosis: an iron-dependent form of nonapoptotic cell death. Cell 149 (5), 1060–1072. 10.1016/j.cell.2012.03.042 22632970 PMC3367386

[B25] Dixon S. J. Stockwell B. R. (2014). The role of iron and reactive oxygen species in cell death. Nat. Chem. Biol. 10 (1), 9–17. 10.1038/nchembio.1416 24346035

[B26] Dodson M. Castro-Portuguez R. Zhang D. D. (2019). NRF2 plays a critical role in mitigating lipid peroxidation and ferroptosis. Redox Biol. 23, 101107. 10.1016/j.redox.2019.101107 30692038 PMC6859567

[B27] Du C. Ma C. Geng R. Y. Wang X. M. Wang X. L. Yang J. H. (2025). Bruceine A inhibits TGF-β1/Smad pathway in pulmonary fibrosis by blocking gal3/TGF-β1 interaction. Phytomedicine 136, 156267. 10.1016/j.phymed.2024.156267 39615217

[B28] Eldeen N. E. Moustafa Y. M. Alwaili M. A. Alrehaili A. A. Khodeer D. M. (2023). Synergistic power of piceatannol And/Or vitamin D in bleomycin-induced pulmonary fibrosis *in vivo:* a preliminary study. Biomedicines 11 (10), 16. 10.3390/biomedicines11102647 PMC10604873 37893021

[B29] El Tabaa M. M. El Tabaa M. M. Elgharabawy R. M. Abdelhamid W. G. (2023). Suppressing NLRP3 activation and PI3K/AKT/mTOR signaling ameliorates amiodarone-induced pulmonary fibrosis in rats: a possible protective role of nobiletin. Inflammopharmacology 31 (3), 1373–1386. 10.1007/s10787-023-01168-2 36947298

[B30] Endale H. T. Tesfaye W. Mengstie T. A. (2023). ROS induced lipid peroxidation and their role in ferroptosis. Front. Cell Dev. Biol. 11, 1226044. 10.3389/fcell.2023.1226044 37601095 PMC10434548

[B31] Feng F. F. Cheng P. Xu S. H. Li N. N. Wang H. Zhang Y. (2020). Tanshinone IIA attenuates silica-induced pulmonary fibrosis *via* Nrf2-mediated inhibition of EMT and TGF-β1/Smad signaling. Chemico-Biological Interact. 319, 109024. 10.1016/j.cbi.2020.109024 32097614

[B32] Feng F. F. Cheng P. Zhang H. N. Li N. N. Qi Y. X. Wang H. (2019). The protective role of tanshinone IIA in silicosis rat model *via* TGF-β1/Smad signaling suppression, NOX4 inhibition and Nrf2/ARE signaling activation. Drug Des. Dev. Ther. 13, 4275–4290. 10.2147/dddt.S230572 PMC6930391 31908414

[B33] Feng J. K. Liu H. Jiang K. W. Gong X. Y. Huang R. Zhou C. (2024). Enhanced oxidative stress aggravates BLM-Induced pulmonary fibrosis by promoting cellular senescence through enhancing NLRP3 activation. Life Sci. 358, 123128. 10.1016/j.lfs.2024.123128 39393575

[B34] Gao C. Chang H. Wang Z. X. Jia M. Li Q. Li X. (2023). The mechanism of qingwen gupi decoction on pulmonary fibrosis based on metabolomics and intestinal flora. J. Appl. Microbiol. 134 (1), lxac035. 10.1093/jambio/lxac035 36626779

[B35] Gao M. H. Monian P. Pan Q. H. Zhang W. Xiang J. Jiang X. J. (2016). Ferroptosis is an autophagic cell death process. Cell Res. 26 (9), 1021–1032. 10.1038/cr.2016.95 27514700 PMC5034113

[B36] Ge C. Huang M. S. Han Y. H. Shou C. Li D. Y. Zhang Y. B. (2024). Demethyleneberberine alleviates pulmonary fibrosis through disruption of USP11 deubiquitinating GREM1. Pharmaceuticals 17 (3), 279. 10.3390/ph17030279 38543064 PMC10974697

[B37] Gungor H. Ekici M. Karayigit M. O. Turgut N. H. Kara H. Arslanbas E. (2020). Zingerone ameliorates oxidative stress and inflammation in bleomycin-induced pulmonary fibrosis: modulation of the expression of TGF-β1 and iNOS. Naunyn-Schmiedebergs Archives Pharmacol. 393 (9), 1659–1670. 10.1007/s00210-020-01881-7 32377772

[B38] Guo Y. Zhu M. Y. Shen R. L. (2023). CD5L deficiency protects mice against bleomycin-induced pulmonary fibrosis. Front. Bioscience-Landmark 28 (9), 209. 10.31083/j.fbl2809209 37796694

[B39] Hecker L. Cheng J. Thannickal V. J. (2012). Targeting NOX enzymes in pulmonary fibrosis. Cell. Mol. Life Sci. 69 (14), 2365–2371. 10.1007/s00018-012-1012-7 22618245 PMC3710124

[B40] Hu Y. X. Huang Y. Zong L. J. Lin J. X. Liu X. Ning S. P. (2024). Emerging roles of ferroptosis in pulmonary fibrosis: current perspectives, opportunities and challenges. Cell Death Discov. 10 (1), 301. 10.1038/s41420-024-02078-0 38914560 PMC11196712

[B41] Hua Q. Z. Ren L. (2024). The SIRT1/Nrf2 signaling pathway mediates the anti-pulmonary fibrosis effect of liquiritigenin. Chin. Med. 19 (1), 12. 10.1186/s13020-024-00886-1 38238857 PMC10795230

[B42] Huai B. Ding J. Y. (2020). Atractylenolide III attenuates bleomycin-induced experimental pulmonary fibrosis and oxidative stress in rat model *via* Nrf2/NQO1/HO-1 pathway activation. Immunopharmacol. Immunotoxicol. 42 (5), 436–444. 10.1080/08923973.2020.1806871 32762376

[B43] Huang J. Z. Tong X. Zhang L. Zhang Y. Wang L. Wang D. G. (2020). Hyperoside attenuates bleomycin-induced pulmonary fibrosis development in mice. Front. Pharmacol. 11, 550955. 10.3389/fphar.2020.550955 33192501 PMC7642689

[B44] Jain N. Bhushan B. L. S. Natarajan M. Mehta R. Saini D. K. Chatterjee K. (2024). Advanced 3D *in vitro* lung fibrosis models: contemporary status, clinical uptake, and prospective outlooks. Acs Biomaterials Sci. and Eng. 10 (3), 1235–1261. 10.1021/acsbiomaterials.3c01499 38335198

[B45] Jia L. Sun P. Gao H. Shen J. Gao Y. Meng C. (2019). Mangiferin attenuates bleomycin-induced pulmonary fibrosis in mice through inhibiting TLR4/p65 and TGF-β1/Smad2/3 pathway. J. Pharm. Pharmacol. 71 (6), 1017–1028. 10.1111/jphp.13077 30847938

[B46] Jiang X. J. Stockwell B. R. Conrad M. (2021). Ferroptosis: mechanisms, biology and role in disease. Nat. Rev. Mol. Cell Biol. 22 (4), 266–282. 10.1038/s41580-020-00324-8 33495651 PMC8142022

[B47] Jiang Y. S. Huang C. R. Chen C. S. Jan J. S. (2024). GSH/pH-Sensitive Poly(glycerol sebacate dithiodiglycolate) nanoparticle as a ferroptotic inducer through cooperation with Fe^3+^&gt. Acs Appl. Polym. Mater. 6 (2), 1129–1140. 10.1021/acsapm.3c01770

[B48] Kagan V. E. Mao G. W. Qu F. Angeli J. P. F. Doll S. St Croix C. (2017). Oxidized arachidonic and adrenic PEs navigate cells to ferroptosis. Nat. Chem. Biol. 13 (1), 81–90. 10.1038/nchembio.2238 27842066 PMC5506843

[B49] Karkale S. Khurana A. Saifi M. A. Godugu C. Talla V. (2018). Andrographolide ameliorates silica induced pulmonary fibrosis. Int. Immunopharmacol. 62, 191–202. 10.1016/j.intimp.2018.07.012 30015239

[B50] Khawas S. Dhara T. K. Sharma N. (2024). Efficacy of umbelliferone-loaded nanostructured lipid carrier in the management of bleomycin-induced idiopathic pulmonary fibrosis: experimental and network pharmacology insight. Naunyn-Schmiedebergs Archives Pharmacol. 16, 7171–7186. 10.1007/s00210-024-03744-x 39718612

[B51] Kliment C. R. Oury T. D. (2010). Oxidative stress, extracellular matrix targets, and idiopathic pulmonary fibrosis. Free Radic. Biol. Med. 49 (5), 707–717. 10.1016/j.freeradbiomed.2010.04.036 20452419 PMC13063126

[B52] Kseibati M. O. Sharawy M. H. Salem H. A. (2020). Chrysin mitigates bleomycin-induced pulmonary fibrosis in rats through regulating inflammation, oxidative stress, and hypoxia. Int. Immunopharmacol. 89, 107011. 10.1016/j.intimp.2020.107011 33045575

[B53] Larki-Harchegani A. Fayazbakhsh F. Nourian A. Nili-Ahmadabadi A. (2023). Chlorogenic acid protective effects on paraquat-induced pulmonary oxidative damage and fibrosis in rats. J. Biochem. Mol. Toxicol. 37 (7), e23352. 10.1002/jbt.23352 37010041

[B54] Lee H. L. Kim J. M. Go M. J. Kim T. Y. Joo S. G. Kim J. H. (2023). Protective effect of *Lonicera japonica* on PM_2.5_-Induced pulmonary damage in BALB/c mice *via* the TGF-β and NF-κB pathway. Antioxidants 12 (4), 968. 10.3390/antiox12040968 37107342 PMC10135714

[B55] Li H. X. Wu M. Y. Guo C. Y. Zhai R. Chen J. (2022). Tanshinone IIA regulates Keap1/Nrf2 signal pathway by activating Sestrin2 to restrain pulmonary fibrosis. Am. J. Chin. Med. 50 (8), 2125–2151. 10.1142/s0192415x22500914 36309810

[B56] Li J. Zhou Y. Shu T. Lei W. Tang Q. Yang Y. (2025). Differentiation of lung tissue-resident c-Kit+ cells into microvascular endothelial cells alleviates pulmonary vascular remodeling. Dev. cell 60, 1601–1617.e7. 10.1016/j.devcel.2025.01.010 39909047

[B57] Li J. P. Liu J. Yue W. F. Xu K. Cai W. P. Cui F. (2020). Andrographolide attenuates epithelial-mesenchymal transition induced by TGF-β1 in alveolar epithelial cells. J. Cell. Mol. Med. 24 (18), 10501–10511. 10.1111/jcmm.15665 32705806 PMC7521220

[B58] Li L. Jin R. J. Ji L. (2023a). Pachymic acid ameliorates bleomycin-induced pulmonary fibrosis through inhibiting endoplasmic reticulum stress in rats. Environ. Toxicol. 9, 5382–5390. 10.1002/tox.23824 37163307

[B59] Li R. J. Wu C. Y. Ke H. L. Wang X. P. Zhang Y. W. (2023b). Qing fei hua xian decoction ameliorates bleomycin-induced pulmonary fibrosis by suppressing oxidative stress through balancing ACE-AngII-AT1R/ACE2-Ang-(1-7)-Mas axis. Iran. J. Basic Med. Sci. 26 (1), 107–113. 10.22038/ijbms.2022.67042.14700 36594067 PMC9790060

[B60] Li S. X. Shao L. L. Fang J. G. Zhang J. Chen Y. Q. Yeo A. J. (2021a). Hesperetin attenuates silica-induced lung injury by reducing oxidative damage and inflammatory response. Exp. Ther. Med. 21 (4), 297. 10.3892/etm.2021.9728 33717240 PMC7885076

[B61] Li S. X. Zhang H. M. Chang J. Li D. M. Cao P. X. (2021b). Iron overload and mitochondrial dysfunction orchestrate pulmonary fibrosis. Eur. J. Pharmacol. 912, 174613. 10.1016/j.ejphar.2021.174613 34740581

[B62] Li X. J. Zhang F. Shen A. J. Hu J. Chen M. J. Yang H. Q. (2023c). Peimine alleviated bleomycin-induced pulmonary fibrosis in mice through reducing epithelial-mesenchymal transition, inflammation and oxidative stress and regulating host metabolism. Nat. Product. Commun. 18 (11). 10.1177/1934578x231214947

[B63] Liang D. G. Feng Y. Zandkarimi F. Wang H. Zhang Z. D. Kim J. (2023). Ferroptosis surveillance independent of GPX4 and differentially regulated by sex hormones. Cell 186(13)**,** 2748–2764.e22. 10.1016/j.cell.2023.05.003 37267948 PMC10330611

[B64] Liang D. G. Minikes A. M. Jiang X. J. (2022). Ferroptosis at the intersection of lipid metabolism and cellular signaling. Mol. Cell 82 (12), 2215–2227. 10.1016/j.molcel.2022.03.022 35390277 PMC9233073

[B65] Liu B. Rong Y. M. Sun D. Li W. W. Chen H. Cao B. (2019). Costunolide inhibits pulmonary fibrosis *via* regulating NF-kB and TGF-β_1_/Smad_2_/Nrf_2_-NOX_4_ signaling pathways. Biochem. Biophysical Res. Commun. 510 (2), 329–333. 10.1016/j.bbrc.2019.01.104 30709583

[B66] Liu B. Yang J. Y. Hao J. T. Xie H. F. Shimizu K. Li R. S. (2021). Natural product mogrol attenuates bleomycin-induced pulmonary fibrosis development through promoting AMPK activation. J. Funct. Foods 77, 104280. 10.1016/j.jff.2020.104280

[B67] Liu M. Xu H. Y. Zhang L. Zhang C. Yang L. C. Ma E. L. (2018a). Salvianolic acid B inhibits myofibroblast transdifferentiation in experimental pulmonary fibrosis *via* the up-regulation of Nrf2. Biochem. Biophysical Res. Commun. 495 (1), 325–331. 10.1016/j.bbrc.2017.11.014 29108993

[B68] Liu Q. M. Shi X. G. Tang L. Y. Xu W. H. Jiang S. Ding W. F. (2018b). Salvianolic acid B attenuates experimental pulmonary inflammation by protecting endothelial cells against oxidative stress injury. Eur. J. Pharmacol. 840, 9–19. 10.1016/j.ejphar.2018.09.030 30273543

[B69] Liu Y. Tang A. M. Liu M. Xu C. J. Cao F. Yang C. F. (2024). Tuberostemonine May enhance the function of the SLC7A11/glutamate antiporter to restrain the ferroptosis to alleviate pulmonary fibrosis. J. Ethnopharmacol. 318, 116983. 10.1016/j.jep.2023.116983 37532076

[B70] Liu Y. L. Chen B. Y. Nie J. Zhao G. H. Zhuo J. Y. Yuan J. (2020). Polydatin prevents bleomycin-induced pulmonary fibrosis by inhibiting the TGF-β/Smad/ERK signaling pathway. Exp. Ther. Med. 20 (5), 62. 10.3892/etm.2020.9190 32952652 PMC7485305

[B71] Loo C. Y. Lee W. H. (2022). Nanotechnology-based therapeutics for targeting inflammatory lung diseases. Nanomedicine 17 (12), 865–879. 10.2217/nnm-2021-0447 35315290

[B72] Ma J. Li G. Wang H. Mo C. H. (2024). Comprehensive review of potential drugs with anti-pulmonary fibrosis properties. Biomed. and Pharmacother. 173, 116282. 10.1016/j.biopha.2024.116282 38401514

[B73] Maher T. M. Bendstrup E. Dron L. Langley J. Smith G. Khalid J. M. (2021). Global incidence and prevalence of idiopathic pulmonary fibrosis. Respir. Res. 22 (1), 197. 10.1186/s12931-021-01791-z 34233665 PMC8261998

[B74] McDonough J. E. Martens D. S. Tanabe N. Ahangari F. Verleden S. E. Maes K. (2018). A role for telomere length and chromosomal damage in idiopathic pulmonary fibrosis. Respir. Res. 19, 132. 10.1186/s12931-018-0838-4 29986708 PMC6038197

[B75] Mehrabani M. Goudarzi M. Mehrzadi S. Siahpoosh A. Mohammadi M. Khalili H. (2020). Crocin: a protective natural antioxidant against pulmonary fibrosis induced by bleomycin. Pharmacol. Rep. 72 (4), 992–1001. 10.1007/s43440-019-00023-y 31997260

[B76] Mehrzadi S. Hosseini P. Mehrabani M. Siahpoosh A. Goudarzi M. Khalili H. (2021). Attenuation of bleomycin-induced pulmonary fibrosis in wistar rats by combination treatment of two natural phenolic compounds: Quercetin and gallic acid. Nutr. Cancer-an Int. J. 73 (10), 2039–2049. 10.1080/01635581.2020.1820053 32933341

[B77] Meyer K. C. (2017). Pulmonary fibrosis, part I: epidemiology, pathogenesis, and diagnosis. Expert Rev. Respir. Med. 11 (5), 343–359. 10.1080/17476348.2017.1312346 28345383

[B78] Neves J. Leitz D. Kraut S. Brandenberger C. Agrawal R. Weissmann N. (2017). Disruption of the hepcidin/ferroportin regulatory system causes pulmonary iron overload and restrictive lung disease. Ebiomedicine 20, 230–239. 10.1016/j.ebiom.2017.04.036 28499927 PMC5478206

[B79] Ning X. Zhao W. D. Wu Q. Y. Wang C. L. Liang S. X. (2024). Therapeutic potential of dihydroartemisinin in mitigating radiation-induced lung injury: inhibition of ferroptosis through Nrf2/HO-1 pathways in mice. Immun. Inflamm. Dis. 12 (2), e1175. 10.1002/iid3.1175 38415919 PMC10839538

[B80] Ogger P. P. Byrne A. J. (2020). Lung fibrosis enters the Iron Age. J. Pathology 252 (1), 1–3. 10.1002/path.5489 32510612

[B81] Pallante P. Malapelle U. Nacchio M. Sgariglia R. Galati D. Capitelli L. (2021). Liquid biopsy is a promising tool for genetic testing in idiopathic pulmonary fibrosis. Diagnostics 11 (7), 1202. 10.3390/diagnostics11071202 34359285 PMC8305941

[B82] Pan B. Wu F. P. Lu S. M. Lu W. W. Cao J. H. Cheng F. (2024). Luteolin-loaded hyaluronidase nanoparticles with deep tissue penetration capability for idiopathic pulmonary fibrosis treatment. Small Methods 9, e2400980. 10.1002/smtd.202400980 39370583

[B83] Park S. J. Kim T. H. Lee K. Kang M. A. Jang H. J. Ryu H. W. (2021). Kurarinone attenuates BLM-induced pulmonary fibrosis *via* inhibiting TGF-β signaling pathways. Int. J. Mol. Sci. 22 (16), 8388. 10.3390/ijms22168388 34445094 PMC8395032

[B84] Pei Z. Qin Y. F. Fu X. H. Yang F. F. Huo F. Liang X. (2022). Inhibition of ferroptosis and iron accumulation alleviates pulmonary fibrosis in a bleomycin model. Redox Biol. 57, 102509. 10.1016/j.redox.2022.102509 36302319 PMC9614651

[B85] Peng L. Wen L. Shi Q. F. Gao F. Huang B. Wang C. M. (2021). Chelerythrine ameliorates pulmonary fibrosis *via* activating the Nrf2/ARE signaling pathway. Cell Biochem. Biophysics 79 (2), 337–347. 10.1007/s12013-021-00967-0 33580396

[B86] Peng L. Y. An L. Sun N. Y. Ma Y. Zhang X. W. Liu W. H. (2019). *Salvia miltiorrhiza* restrains reactive oxygen species-associated pulmonary fibrosis *via* targeting Nrf2-Nox4 redox balance. Am. J. Chin. Med. 47 (5), 1113–1131. 10.1142/s0192415x19500575 31352786

[B87] Pramanik S. Mohanto S. Manne R. Rajendran R. R. Deepak A. Edapully S. J. (2021). Nanoparticle-based drug delivery system: the magic bullet for the treatment of chronic pulmonary diseases. Mol. Pharm. 18 (10), 3671–3718. 10.1021/acs.molpharmaceut.1c00491 34491754

[B88] Qu J. Zhu L. Y. Zhou Z. J. Chen P. Liu S. Y. Locy M. L. (2018). Reversing mechanoinductive DSP expression by CRISPR/dCas9-mediated epigenome editing. Am. J. Respir. Crit. Care Med. 198 (5), 599–609. 10.1164/rccm.201711-2242OC 29924937 PMC6118013

[B89] Raish M. Ahmad A. Ansari M. A. Ahad A. Al-Jenoobi F. I. Al-Mohizea A. M. (2018). Sinapic acid ameliorates bleomycin-induced lung fibrosis in rats. Biomed. and Pharmacother. 108, 224–231. 10.1016/j.biopha.2018.09.032 30219680

[B90] Ren G. Q. Xu G. H. Li R. S. Xie H. F. Cui Z. G. Wang L. (2023). Modulation of bleomycin-Induced oxidative stress and pulmonary fibrosis by ginkgetin in mice *via* AMPK. Curr. Mol. Pharmacol. 16 (2), 217–227. 10.2174/1874467215666220304094058 35249515

[B91] Rong Y. M. Cao B. Liu B. Li W. W. Chen Y. Z. Chen H. (2018). A novel Gallic acid derivative attenuates BLM-Induced pulmonary fibrosis in mice. Int. Immunopharmacol. 64, 183–191. 10.1016/j.intimp.2018.08.024 30195109

[B92] Savin I. A. Zenkova M. A. Sen'kova A. V. (2022). Pulmonary fibrosis as a result of acute lung inflammation: molecular mechanisms, relevant *in vivo* models, prognostic and therapeutic approaches. Int. J. Mol. Sci. 23 (23), 42. 10.3390/ijms232314959 PMC9735580 36499287

[B93] Shao M. Cheng H. P. Li X. H. Qiu Y. J. Zhang Y. N. Chang Y. F. (2024). Abnormal mitochondrial iron metabolism damages alveolar type II epithelial cells involved in bleomycin-induced pulmonary fibrosis. Theranostics 14 (7), 2687–2705. 10.7150/thno.94072 38773980 PMC11103499

[B94] Shariati S. Kalantar H. Pashmforoosh M. Mansouri E. Khodayar M. J. (2019). Epicatechin protective effects on bleomycin-induced pulmonary oxidative stress and fibrosis in mice. Biomed. and Pharmacother. 114, 108776. 10.1016/j.biopha.2019.108776 30903918

[B95] Shen X. B. Ding D. L. Yu L. Z. Ni J. Z. Liu Y. Wang W. (2022). Total extract of anemarrhenae rhizoma attenuates bleomycin-induced pulmonary fibrosis in rats. Bioorg. Chem. 119, 105546. 10.1016/j.bioorg.2021.105546 34954573

[B96] Sherekar P. Suke S. G. Dhok A. Harode R. Mangrulkar S. Pingle S. (2024). Nano-enabled delivery of diosgenin and emodin ameliorates respirable silica dust-induced pulmonary fibrosis silicosis in rats. Ecotoxicol. Environ. Saf. 279, 116483. 10.1016/j.ecoenv.2024.116483 38788565

[B97] Shi W. Y. Feng B. Xu S. G. Shen X. Y. Zhang T. F. (2017). Inhibitory effect of compound chuanxiong kangxian granules on bleomycin-induced pulmonary fibrosis in rats. Biomed. and Pharmacother. 96, 1179–1185. 10.1016/j.biopha.2017.11.104 29239818

[B98] Sun L. F. He X. X. Kong J. Zhou J. Y. (2024a). Protection of qingfei xieding prescription from idiopathic pulmonary fibrosis by regulating renin-angiotensin and ferroptosis in MLE-12 cells. Histology Histopathol. 39 (12), 1643–1658. 10.14670/hh-18-746 38666295

[B99] Sun Y. Ren Y. Song L. Y. Wang Y. Y. Wu Y. L. Li L. (2024b). Targeting iron-metabolism:a potential therapeutic strategy for pulmonary fibrosis. Biomed. and Pharmacother. 172, 116270. 10.1016/j.biopha.2024.116270 38364737

[B100] Tao L. J. Cao J. Wei W. C. Xie H. F. Zhang M. Zhang C. F. (2017). Protective role of rhapontin in experimental pulmonary fibrosis *in vitro* and *in vivo* i&g. Int. Immunopharmacol. 47, 38–46. 10.1016/j.intimp.2017.03.020 28364627

[B101] Tian H. Wang L. M. Fu T. L. (2023). Ephedrine alleviates bleomycin-induced pulmonary fibrosis by inhibiting epithelial-mesenchymal transition and restraining NF-κB signaling. J. Toxicol. Sci. 48 (10), 547–556. 10.2131/jts.48.547 37778983

[B102] Tian S. L. Yang Y. Liu X. L. Xu Q. B. (2018). Emodin attenuates bleomycin-induced pulmonary fibrosis *via* anti-inflammatory and anti-oxidative activities in rats. Med. Sci. Monit. 24, 1–10. 10.12659/msm.905496 29290631 PMC5759514

[B103] Tomas G. Elizabeta N. (2015). Iron homeostasis in host defence and inflammation. Nat. Rev. Immunol. 15 (8), 500–510. 10.1038/nri3863 26160612 PMC4801113

[B104] Veith C. Drent M. Bast A. van Schooten F. J. Boots A. W. (2017). The disturbed redox-balance in pulmonary fibrosis is modulated by the plant flavonoid quercetin. Toxicol. Appl. Pharmacol. 336, 40–48. 10.1016/j.taap.2017.10.001 28987380

[B105] Veith C. Hristova M. Danyal K. Habibovic A. Dustin C. M. McDonough J. E. (2021). Profibrotic epithelial TGF-β1 signaling involves NOX4-mitochondria cross talk and redox-mediated activation of the tyrosine kinase FYN. Am. J. Physiology-Lung Cell. Mol. Physiology 320 (3), L356–L367. 10.1152/ajplung.00444.2019 PMC8354818 33325804

[B106] Verma S. Dutta A. Dahiya A. Kalra N. (2022). Quercetin-3-Rutinoside alleviates radiation-induced lung inflammation and fibrosis *via* regulation of NF-ΚB/TGF-β1 signaling. Phytomedicine 99, 154004. 10.1016/j.phymed.2022.154004 35219007

[B107] von Krusenstiern A. N. Robson R. N. Qian N. X. Qiu B. Y. Hu F. H. Reznik E. (2023). Identification of essential sites of lipid peroxidation in ferroptosis. Nat. Chem. Biol. 19(6)**,** 719–730. 10.1038/s41589-022-01249-3 36747055 PMC10238648

[B108] Wan Q. Y. Zhang X. R. Zhou D. F. Xie R. Cai Y. Zhang K. H. (2023). Inhaled nano-based therapeutics for pulmonary fibrosis: recent advances and future prospects. J. Nanobiotechnology 21 (1), 215. 10.1186/s12951-023-01971-7 37422665 PMC10329312

[B109] Wang L. Shao M. Jiang W. Huang Y. F. (2022). Resveratrol alleviates bleomycin-induced pulmonary fibrosis by inhibiting epithelial-mesenchymal transition and down-regulating TLR4/NF-ΚB and TGF-β1/smad3 signalling pathways in rats. Tissue and Cell 79, 101953. 10.1016/j.tice.2022.101953 36228366

[B110] Wang S. M. Tan W. Zhang L. Jiang H. B. (2023). Pachymic acid protects against bleomycin-induced pulmonary fibrosis by suppressing fibrotic, inflammatory, and oxidative stress pathways in mice. Appl. Biochem. Biotechnol. 12, 3344–3355. 10.1007/s12010-023-04686-5 37650950

[B111] Wang Y. Dong X. M. Zhao N. Su X. M. Wang Y. Y. Li Y. F. (2020). Schisandrin B attenuates bleomycin-induced pulmonary fibrosis in mice through the wingless/integrase-1 signaling pathway. Exp. Lung Res. 46 (6), 185–194. 10.1080/01902148.2020.1760964 32362157

[B112] Wei Y. J. Ni W. T. Zhao L. Z. Gao Y. H. Zhou B. Feng Q. (2025). Phillygenin inhibits PI3K-Akt-mTOR signalling pathway to prevent bleomycin-induced idiopathic pulmonary fibrosis in mice. Clin. Exp. Pharmacol. Physiology 52 (2), e70017. 10.1111/1440-1681.70017 39746665

[B113] Wen J. Wang C. Song L. Y. Wang Y. Y. Liang P. T. Pang W. L. (2024). Ferroptosis mediates pulmonary fibrosis: implications for the effect of astragalus and Panax notoginseng decoction. Can. Respir. J. 18, 5554886. 10.1155/2024/5554886 PMC10997418 38584671

[B114] Wiernicki B. Dubois H. Tyurina Y. Y. Hassannia B. Bayir H. Kagan V. E. (2020). Excessive phospholipid peroxidation distinguishes ferroptosis from other cell death modes including pyroptosis. Cell Death and Dis. 11 (10), 922. 10.1038/s41419-020-03118-0 PMC7591475 33110056

[B115] Winterbourn C. C. (2008). Reconciling the chemistry and biology of reactive oxygen species. Nat. Chem. Biol. 4 (5), 278–286. 10.1038/nchembio.85 18421291

[B116] Xin X. B. Yao D. H. Zhang K. Han S. Liu D. N. Wang H. Y. (2019). Protective effects of rosavin on bleomycin-induced pulmonary fibrosis *via* suppressing fibrotic and inflammatory signaling pathways in mice. Biomed. and Pharmacother. 115, 108870. 10.1016/j.biopha.2019.108870 31026730

[B117] Xu M. J. Zhang D. Yan J. (2024). Targeting ferroptosis using Chinese herbal compounds to treat respiratory diseases. Phytomedicine 130, 155738. 10.1016/j.phymed.2024.155738 38824825

[B118] Xu W. T. Deng H. M. Hu S. Zhang Y. G. Zheng L. Liu M. Y. (2021). Role of ferroptosis in lung diseases. J. Inflamm. Res. 14, 2079–2090. 10.2147/jir.S307081 34045882 PMC8144020

[B119] Yan L. Song F. Li H. Li Y. Li J. He Q. Y. (2018). Submicron emulsion of cinnamaldehyde ameliorates bleomycin-induced idiopathic pulmonary fibrosis *via* inhibition of inflammation, oxidative stress and epithelial-mesenchymal transition. Biomed. and Pharmacother. 102, 765–771. 10.1016/j.biopha.2018.03.145 29604596

[B120] Yang D. X. Qiu J. Zhou H. H. Yu Y. Zhou D. L. Xu Y. (2018). Dihydroartemisinin alleviates oxidative stress in bleomycin-induced pulmonary fibrosis. Life Sci. 205, 176–183. 10.1016/j.lfs.2018.05.022 29752961

[B121] Yang H. H. Wang L. D. Yang M. S. Hu J. Q. Zhang E. L. Peng L. P. (2022a). Oridonin attenuates LPS-Induced early pulmonary fibrosis by regulating impaired autophagy, oxidative stress, inflammation and EMT. Eur. J. Pharmacol. 923, 174931. 10.1016/j.ejphar.2022.174931 35398392

[B122] Yang L. Cao L. M. Zhang X. J. Chu B. (2022b). Targeting ferroptosis as a vulnerability in pulmonary diseases. Cell Death and Dis. 13 (7), 649. 10.1038/s41419-022-05070-7 PMC9315842 35882850

[B123] Yao J. J. Li Y. X. Meng F. Shen W. W. Wen H. (2023). Enhancement of suppression oxidative stress and inflammation of quercetin by nano-decoration for ameliorating silica-induced pulmonary fibrosis. Environ. Toxicol. 38 (7), 1494–1508. 10.1002/tox.23781 37017410

[B124] Yuan L. Y. Sun Y. Zhou N. Wu W. P. Zheng W. D. Wang Y. K. (2022). Dihydroquercetin attenuates silica-induced pulmonary fibrosis by inhibiting ferroptosis signaling pathway. Front. Pharmacol. 13, 845600. 10.3389/fphar.2022.845600 35645837 PMC9133504

[B125] Zaghloul M. S. Said E. Suddek G. M. Salem H. A. (2019). Crocin attenuates lung inflammation and pulmonary vascular dysfunction in a rat model of bleomycin-induced pulmonary fibrosis. Life Sci. 235, 116794. 10.1016/j.lfs.2019.116794 31465731

[B126] Zeng Q. Wen B. B. Liu X. Luo Y. Y. Hu Z. G. Huang L. (2024). NBR1-p62-Nrf2 mediates the anti-pulmonary fibrosis effects of protodioscin. Chin. Med. 19 (1), 60. 10.1186/s13020-024-00930-0 38589903 PMC11003024

[B127] Zhai X. R. Zhu J. Y. Li J. Wang Z. X. Zhang G. F. Nie Y. J. (2023). Fraxetin alleviates BLM-Induced idiopathic pulmonary fibrosis by inhibiting NCOA4-mediated epithelial cell ferroptosis. Inflamm. Res. 72 (10-11), 1999–2012. 10.1007/s00011-023-01800-5 37798541

[B128] Zhang D. Liu B. Cao B. Wei F. Yu X. Li G. F. (2017). Synergistic protection of schizandrin B and glycyrrhizic acid against bleomycin-induced pulmonary fibrosis by inhibiting TGF-β1/Smad2 pathways and overexpression of NOX4. Int. Immunopharmacol. 48, 67–75. 10.1016/j.intimp.2017.04.024 28476015

[B129] Zhang F. Xiang Y. Ma Q. Guo E. Zeng X. S. (2024a). A deep insight into ferroptosis in lung disease: facts and perspectives. Front. Oncol. 14, 1354859. 10.3389/fonc.2024.1354859 38562175 PMC10982415

[B130] Zhang T. Sun C. Yang S. B. Cai Z. M. Zhu S. F. Liu W. D. (2024b). Inhalation of taraxasterol loaded mixed micelles for the treatment of idiopathic pulmonary fibrosis. Chin. Chem. Lett. 35 (8), 109248. 10.1016/j.cclet.2023.109248

[B131] Zhao H. Li C. D. Li L. N. Liu J. Y. Gao Y. H. Mu K. (2020). Baicalin alleviates bleomycin-induced pulmonary fibrosis and fibroblast proliferation in rats *via* the PI3K/AKT signaling pathway. Mol. Med. Rep. 21 (6), 2321–2334. 10.3892/mmr.2020.11046 32323806 PMC7185294

[B132] Zheng M. L. Liu K. Li L. Feng C. L. Wu G. H. (2024). Traditional Chinese medicine inspired dual-drugs loaded inhalable nano-therapeutics alleviated idiopathic pulmonary fibrosis by targeting early inflammation and late fibrosis. J. Nanobiotechnology 22 (1), 14. 10.1186/s12951-023-02251-0 38166847 PMC10763202

[B133] Zhou Z. Kandhare A. D. Kandhare A. A. Bodhankar S. L. (2019). Hesperidin ameliorates bleomycin-induced experimental pulmonary fibrosis *via* inhibition of TGF-beta1/Smad3/AMPK and IkappaBalpha/NF-kappaB pathways. Excli J. 18, 723–745. 10.17179/excli2019-1094 31611754 PMC6785776

[B134] Zhu J. Q. Tian Y. Y. Chan K. L. Hu Z. Xu Q. Q. Lin Z. X. (2024). Modified qing-zao-jiu-fei decoction attenuated pulmonary fibrosis induced by bleomycin in rats *via* modulating Nrf2/NF-κB and MAPKs pathways. Chin. Med. 19 (1), 10. 10.1186/s13020-024-00882-5 38229198 PMC10790405

